# Association between injury severity and recovery of objective functional measures following adolescent lateral ankle sprain: a secondary cohort analysis

**DOI:** 10.3389/fspor.2026.1966025

**Published:** 2026-10-08

**Authors:** Matas Meškauskas, Saulius Rutkauskas, Miglė Misiūnienė, Jūratė Tomkevičiūtė, Paulina Kriauzaitė, Dalius Malcius, Emilis Čekanauskas

**Affiliations:** 1 Department of Pediatric Surgery, Lithuanian University of Health Sciences, Kaunas, Lithuania; 2 Department of Radiology, Lithuanian University of Health Sciences, Kaunas, Lithuania; 3 Department of Sports Medicine, Lithuanian University of Health Sciences, Kaunas, Lithuania; 4 Department of Physics, Mathematics and Biophysics, Lithuanian University of Health Sciences, Kaunas, Lithuania; 5 Medical Academy, Lithuanian University of Health Sciences, Kaunas, Lithuania

**Keywords:** acute lateral ankle ligament injury, biodex, grade III lateral ankle ligament tear, lateral ankle sprain, Y balance test

## Abstract

**Background/objectives:**

Lateral ankle sprain (LAS) is common in adolescents, but the extent to which structural injury severity is associated with recovery of objective functional measures remains unclear. This study explored the association between Grade I–III injury severity and short-term recovery of objective functional measures following adolescent LAS using a *post hoc* secondary analysis of a randomized controlled trial (RCT).

**Methods:**

This exploratory secondary cohort analysis included 65 adolescents aged 12–17 years with acute LAS who had participated in a RCT comparing two rehabilitation strategies. The severity-based analysis was not prespecified in the trial protocol. Participants were analyzed according to Grade I–III injury severity, irrespective of original treatment allocation. Injury severity was classified using clinical and imaging findings. Functional performance was assessed at 1–2, 5–7, and 12–15 weeks using Biodex isokinetic dynamometry for inversion (IN) and eversion (EV) peak torque normalized to body weight (PEAK TQ/BW) and ankle range of motion (ROM), and the Y-Balance Test composite score (YBT-CS). Outcomes were expressed as signed side-to-side differences (uninjured−injured), with values closer to zero indicating greater interlimb symmetry. Changes were analyzed using mixed repeated-measures ANOVA and covariate-adjusted LMMs including Severity, Time, Severity × Time, age, and treatment allocation, with participant as a random effect. Injured- and uninjured-limb values were examined separately.

**Results:**

Significant changes over time were observed for most outcomes. No significant Severity × Time interactions were identified for signed side-to-side differences in either repeated-measures ANOVA or covariate-adjusted LMMs. In the ANOVA, Severity main effects were observed for ROM at 120°/s (*p* = 0.024) and YBT-CS (*p* = 0.014); after adjustment, the Severity effect remained significant for YBT-CS (*p* = 0.045) but not ROM at 120°/s (*p* = 0.090). In injured-limb analyses, all outcomes improved over time (all *p* < 0.001), without significant Severity or Severity × Time effects.

**Conclusions:**

No statistically significant severity-related differences in short-term recovery trajectories were identified for the measured outcomes, including in covariate-adjusted analyses. The Severity effect for YBT-CS, but not ROM at 120°/s, remained significant after adjustment. Given the exploratory design and limited power for interaction effects, these findings should be considered hypothesis-generating.

**Trial registration:**

NCT07287020; registered 3 December 2025 (retrospectively registered).

## 1 Introduction

Lateral ankle sprain (LAS) is one of the most common musculoskeletal injuries in adolescents participating in sports and physical activities (1). Despite its high incidence, variability remains in clinical presentation, management strategies, and recovery trajectories (2). LAS is associated with one of the highest reinjury rates among lower limb injuries, with approximately a twofold increased risk of recurrence within the first year (3).

Despite being frequently considered a minor injury, LAS may progress to chronic ankle instability (CAI), a condition marked by recurrent sprains, ongoing symptoms, and functional impairment (4). A substantial proportion of individuals experience persistent symptoms following an initial ankle sprain. Previous studies have reported that approximately one-third of patients continue to report pain, swelling, or recurrent sprains several years after injury, with many exhibiting at least one residual symptom affecting daily activities and sports participation. These factors highlight the clinical importance of optimizing early management and understanding factors that influence recovery (5).

Injury severity is commonly classified into Grade I–III based on the extent of ligament damage, typically determined using imaging modalities such as ultrasound or magnetic resonance imaging (MRI) in conjunction with clinical findings, including swelling, hematoma, and signs of mechanical instability (e.g., anterior drawer test) (6). In clinical practice, higher-grade injuries are often perceived as more severe and may lead to more restrictive treatment approaches, including prolonged immobilization and limitation of physical activity. However, the extent to which injury severity is associated with recovery of objective functional measures remains unclear (7, 8).

The identification of early prognostic factors associated with poor recovery may provide an opportunity to guide targeted interventions and improve clinical outcomes (5). Conservative management is widely recommended as the first-line treatment for LAS, with surgical intervention typically reserved for cases of persistent mechanical or functional instability following at least 6 months of structured rehabilitation (1, 9). However, it remains unclear whether injury severity is associated with delayed recovery or whether assumptions regarding higher-grade injuries may lead to unnecessarily restrictive or overly conservative management strategies (10, 11).

Although numerous studies have described the classification of LAS based on ligament injury severity and have evaluated different rehabilitation strategies, the relationship between structural injury grade and subsequent functional recovery remains unclear. Evidence examining associations between injury severity and recovery of objective functional measures is limited, particularly with respect to objective biomechanical outcomes such as dynamic balance and isokinetic strength. In addition, there is a lack of research focusing specifically on adolescent populations (9, 10).

The present study used data from a previously completed randomized controlled trial (RCT) comparing PRICE (Protection, Rest, Ice, Compression, and Elevation) and PEACE and LOVE (Protection, Elevation, Avoidance of anti-inflammatory modalities, Compression, and Education followed by Load, Optimism, Vascularization, and Exercise) rehabilitation strategies (12). In this secondary analysis, participants were examined according to Grade I–III LAS severity to explore whether structural injury grade was associated with differences in the short-term recovery of objective functional measures. Because this severity-based question was not prespecified in the original trial protocol, the analysis was considered exploratory.

## 2 Materials and methods

### 2.1 Study design and participants

This study was an exploratory *post hoc* secondary analysis of data derived from a previously conducted prospective RCT comparing two rehabilitation strategies following acute LAS. The original trial was designed to compare PRICE and PEACE and LOVE rehabilitation approaches, with participants randomized to one of the two treatment groups. These protocols differ primarily in their approach to inflammation management, early loading, and patient education (12). The original RCT was retrospectively registered at ClinicalTrials.gov (NCT07287020; 3 December 2025), after completion of participant recruitment. The severity-based research question was not prespecified in the original trial protocol, trial registration, or a prospective statistical analysis plan. Although the question was discussed informally during the course of the original study, no prospective severity-based analysis plan or protocol amendment was documented. For this secondary analysis, participants were analyzed observationally as a cohort according to injury severity (Grade I–III), rather than according to randomized treatment allocation, to examine the association between structural injury grade and short-term recovery of objective functional measures. The study was reported in accordance with the STROBE guidelines for observational cohort studies.

In the original trial, no statistically significant differences in functional outcomes were observed between treatment groups. For the present exploratory severity-based analysis, participants from both treatment groups were analyzed collectively; original treatment allocation was subsequently included as a covariate in the linear mixed-model sensitivity analyses. However, the absence of a statistically significant treatment effect does not exclude a potential influence of rehabilitation allocation on the present associations, and treatment-related residual confounding is therefore considered when interpreting the findings.

Adolescents aged 12–17 years presenting with a first-time acute LAS were recruited from Lithuanian University of Health Sciences (LUHS) Kaunas Clinics, Department of Pediatric Orthopedics, between February 2022 and January 2025. Inclusion criteria were as follows: (1) first-time LAS, (2) acute injury within 1–4 days of trauma, and (3) absence of chronic ankle pain or fracture, except for minor avulsion fractures. Participants with a history of ankle surgery, systemic disease, or neurological disorders were excluded. A total of 76 participants were enrolled, of whom 65 completed follow-up and were included in the final analysis. Among those excluded, 6 participants did not attend scheduled follow-up visits, 3 experienced re-injury associated with non-adherence to the rehabilitation protocol, and 2 underwent surgical intervention due to osteochondral lesions.

### 2.2 Ethical approval

Ethical approval for the study was granted by the Kaunas Regional Biomedical Research Ethics Committee (Protocol No. 2022-BE10-0003). Written informed consent was obtained from all participants and their legal guardians prior to enrollment.

### 2.3 Injury severity classification

Clinical assessment of LAS was performed by a pediatric orthopedic surgeon. Ultrasound examinations were conducted by a single experienced musculoskeletal radiologist with over 15 years of practice (more than 50,000 musculoskeletal ultrasound examinations), ensuring consistency in imaging evaluation. Diagnosis was established based on clinical findings and confirmed using ultrasound and radiographic imaging (MyLab 9 eXP 20071, Esaote S.p.A., CE 0123).

Injury severity was categorized according to the extent of damage to the lateral ligament complex of the ankle, comprising the anterior talofibular ligament (ATFL), calcaneofibular ligament (CFL), and posterior talofibular ligament (PTFL). Classification was based on a combination of clinical examination and imaging findings, in line with established orthopedic and imaging criteria (8).

Grade I injuries were defined as ligament stretching or microscopic damage without visible fiber disruption, most commonly affecting the ATFL, with preserved structural continuity on ultrasound and no evidence of mechanical instability on clinical examination.

Grade II injuries were characterized by partial tearing of the ATFL, with or without partial involvement of the CFL, demonstrated on ultrasound by ligament thickening, hypoechogenicity, or partial discontinuity of fibers, and clinically associated with moderate pain, swelling, and functional impairment.

Grade III injuries were defined as complete rupture of the ATFL, typically accompanied by injury to the CFL and, in some cases, the PTFL. This category also included avulsion-type injuries, involving detachment of the ligament from its bony insertion, with or without an associated bone fragment, confirmed by imaging. Clinically, these injuries presented with pronounced swelling, ecchymosis, and clear signs of mechanical instability (1, 7, 8, 13, 14). No formal inter-rater or intra-rater reliability analysis was performed for the Grade I–III injury-severity classification.

Injuries involving the distal tibiofibular syndesmosis (high ankle sprains) were considered anatomically and biomechanically distinct from lateral ankle ligament injuries and were therefore excluded from this classification (15).

### 2.4 Outcome measures

Depending on symptom severity, 30 participants (46.2%) received temporary immobilization with a plaster cast in the emergency department. The first outpatient assessment was performed within 1–4 days after the emergency department visit. At this visit, all plaster casts were removed, and all participants, irrespective of whether temporary plaster immobilization had been applied, were provided with a lace-up ankle brace. Thus, plaster immobilization, when used, was limited to the immediate acute post-injury period and was not continued beyond the first outpatient assessment. All participants subsequently followed a conservative rehabilitation protocol (PRICE or PEACE and LOVE).

Recovery of objective functional measures was assessed using biomechanical testing at 1–2 weeks (T1), 5–7 weeks (T2), and 12–15 weeks (T3) after injury. The first assessment was scheduled at 1–2 weeks post-injury to allow for reduction of acute pain and swelling, as early isokinetic testing may be limited by pain and may not reflect maximal muscle performance.

Isokinetic muscle strength and ankle range of motion (ROM) were evaluated using a Biodex System 4 dynamometer (model 835-210, CE 0413, USA). Peak torque normalized to body weight (Peak TQ/BW) was measured during inversion (IN) and eversion (EV) movements. ROM represented the total angular excursion recorded by the dynamometer during the IN–EV movement performed at angular velocities of 60°/s and 120°/s and was expressed in degrees (°). Testing was performed by a single sports medicine physician in collaboration with the Lithuanian University of Health Sciences Department of Sports Medicine to ensure consistency. The Biodex dynamometer demonstrates good to excellent reliability for ankle strength assessment, with reported intraclass correlation coefficients (ICC) ranging from 0.74 to 0.96 (16).

To account for inter-individual variability, all outcomes were expressed as signed side-to-side differences, calculated as the difference between the contralateral uninjured limb and the injured limb (uninjured−injured). Positive values indicate better performance of the uninjured limb, values close to zero indicate greater interlimb symmetry, and negative values indicate that the injured limb performed better than the contralateral limb. Because these were signed differences, movement toward zero rather than a numerically smaller value was interpreted as greater symmetry. The contralateral limb was used as a within-participant comparison limb and should not be considered a true preinjury reference.

To further characterize the changes underlying the signed side-to-side differences, the original measured values for the injured and uninjured limbs were also examined separately at each assessment time point. This allowed changes in interlimb symmetry to be interpreted alongside the corresponding performance of each limb.

Dynamic balance and integrated sensorimotor performance were assessed using the Y-Balance Test (YBT) (Functional Movement Systems, Danville, VA, USA). Participants performed single-leg stance trials while reaching with the contralateral limb in three directions: anterior, posteromedial, and posterolateral. Each direction was tested three times per limb, and the maximum reach distance was recorded. Limb length was measured from the anterior superior iliac spine to the distal tip of the medial malleolus.

The YBT composite score (CS) was calculated by summing the maximal reach distances in all three directions, dividing by three times the limb length, and multiplying by 100. The YBT has demonstrated good reliability and validity in assessing dynamic balance, with reported ICC values ranging from 0.85 to 0.91 (17, 18).

### 2.5 Testing procedure

Prior to testing, all participants were instructed to refrain from vigorous physical activity for 24 h and avoid food intake for at least 2 h. Anthropometric measurements were obtained before assessment. A standardized 5-minute warm-up was performed on a stationary bicycle at 60–70 rpm, 70 W resistance, and a target heart rate of 110–130 bpm. Testing conditions were standardized at 23 °C and 77% relative humidity. After a 5-minute rest period, participants were positioned and stabilized on the Biodex dynamometer using chest, waist, thigh, and calf straps. Familiarization trials were completed at approximately 25%, 50%, 75%, and 100% of perceived maximal effort before formal testing. Participants then performed three maximal repetitions at angular velocities of 60°/s and 120°/s, with 60-second rest intervals between sets. The uninjured limb was consistently tested first, followed by the injured limb. Standardized verbal encouragement was provided throughout testing to promote maximal effort.

The Y-Balance Test was administered according to a standardized protocol. All assessments were performed barefoot to minimize footwear-related variability. Participants maintained the stance foot on the central platform and kept both hands on the hips throughout testing. Standardized instructions, demonstration, and familiarization trials were provided before data collection. Three recorded trials were completed in the anterior, posteromedial, and posterolateral directions for each limb, with short standardized rest intervals between trials and directions. The testing order was identical for all participants, and the uninjured limb was tested first. Trials were repeated if participants lost balance, removed their hands from the hips, displaced the stance foot, or used the reaching limb for support. Only standardized neutral verbal cues were used during testing to ensure consistency between participants (18).

The sports medicine physician performing the Biodex and YBT assessments was blinded to both injury severity and rehabilitation group allocation (PRICE vs. PEACE and LOVE). The radiologist was not involved in the functional outcome assessments and did not have access to the Biodex or YBT results at the time of injury classification.

### 2.6 Statistical analysis

Data were analyzed using IBM SPSS Statistics (version 30.0; IBM Corp., Armonk, NY, USA). All outcomes were treated as continuous variables. The primary outcome representation was the signed side-to-side difference (uninjured−injured), as defined in Section 2.4. In addition, values for the injured and uninjured limbs were summarized separately to characterize the limb-specific changes underlying the side-to-side differences. Continuous variables are presented as mean [standard deviation (SD)] or median [interquartile range (IQR)], depending on data distribution, and categorical variables are presented as frequency (%). Normality of continuous variables was assessed using the Shapiro–Wilk test. Baseline comparability across injury severity groups was assessed using one-way ANOVA for normally distributed variables or the Kruskal–Wallis test for non-normally distributed variables, and chi-square tests for categorical variables.

For the primary analysis of signed side-to-side differences, each functional outcome (Peak TQ/BW in IN and EV, ROM at 60°/s and 120°/s, and YBT composite score) was analyzed using a two-way mixed repeated-measures ANOVA, with Severity (Grade I, Grade II, Grade III) as the between-subject factor and Time (T1: 1–2 weeks; T2: 5–7 weeks; T3: 12–15 weeks) as the within-subject factor. The primary inferential test was the Severity × Time interaction. Effect sizes for the Time, Severity, and Severity × Time effects are reported as partial eta squared (*η*p^2^).

As an additional analysis, injured- and uninjured-limb values were summarized separately at each assessment time point using descriptive statistics to determine whether changes in interlimb symmetry were accompanied by changes in either limb. Injured-limb values were also analyzed longitudinally using the same two-way mixed repeated-measures ANOVA framework, with Severity as the between-subject factor and Time as the within-subject factor. The Severity × Time interaction was used to assess whether changes in injured-limb performance over time differed according to injury severity.

When the interaction was statistically significant, follow-up analyses examined (i) between-group differences at each time point and (ii) within-group changes over time using planned pairwise comparisons based on estimated marginal means. Because these comparisons were hypothesis-driven and contingent on the omnibus interaction test, no Bonferroni adjustment was applied; exact *p* values are reported. When the interaction was not significant, results were interpreted primarily in terms of the main effects of Time and Severity.

Model assumptions were assessed by inspection of residuals. Homogeneity of variances was evaluated using Levene's test at each time point. Sphericity for the within-subject factor (Time) was assessed using Mauchly's test; when violated, the Greenhouse–Geisser correction was applied. Where applicable, 95% confidence intervals (CI) are provided. All tests were two-sided, with statistical significance set at *p* < 0.05. Analyses were conducted per-protocol, including only participants who completed all three assessments. Missing data were not imputed; analyses were restricted to participants with complete data (per-protocol analysis). For the 120°/s outcomes, one participant was excluded from the repeated-measures analyses because data from the first assessment were unavailable due to a technical malfunction of the isokinetic dynamometer; therefore, these analyses included 64 participants.

Seven separate outcome models were examined. Given the exploratory nature of this secondary analysis, no adjustment for multiple comparisons was applied; therefore, *p*-values should be interpreted as nominal and with appropriate caution.

To further evaluate the primary signed side-to-side difference outcomes, covariate-adjusted longitudinal linear mixed-effects models were performed. Severity, Time, Severity × Time, age, and original treatment allocation (PRICE vs. PEACE and LOVE) were included as fixed effects, with participant included as a random effect to account for within-participant repeated measurements. The Severity × Time interaction was the primary effect of interest. Fixed-effect estimates with 95% confidence intervals and *p*-values are reported in the Supplementary Material.

As a sensitivity analysis, injured-limb values were additionally analyzed using longitudinal linear mixed models adjusted for age and original treatment allocation (PRICE vs. PEACE and LOVE). Severity, Time, Severity × Time, age, and treatment allocation were included as fixed effects. Repeated observations within participants were modeled using an unstructured covariance structure, with restricted maximum likelihood estimation and Satterthwaite approximation of degrees of freedom. Models incorporating an additional participant-level random intercept were initially evaluated but did not achieve stable convergence; therefore, the final models used the converged repeated unstructured covariance specification without an additional random intercept. The Severity × Time interaction remained the primary effect of interest. A broader sensitivity analysis additionally adjusted for BMI. Fixed-effect estimates with 95% confidence intervals and omnibus tests of fixed effects are reported in the Supplementary Material.

### 2.7 Power analysis and sample size determination

*A priori* sample size estimation was performed for the original study based on expected changes in IN muscle strength, identified as one of the most affected variables following LAS (19, 20). Based on pilot data collected at the LUHS, a mean side-to-side IN strength difference of 2.65% (SD 7.5%) was assumed. The analysis was conducted using IBM SPSS Statistics, assuming a Pearson correlation of 0.5, a two-tailed significance level of 0.05, and a statistical power of 80%. Under these assumptions, a minimum sample size of 65 participants was required to detect a clinically meaningful change in strength over 12–15 weeks, assuming near-symmetrical strength restoration between the injured and uninjured limbs at the end of the follow-up period.

To account for an anticipated attrition rate of approximately 15%, a total of 76 participants were recruited. The sample-size calculation was performed for the objectives of the original RCT and was not designed specifically to detect differences in recovery trajectories among Grade I, Grade II, and Grade III injury groups. The severity groups in the present analysis were unequal in size (Grade I, *n* = 14; Grade II, *n* = 16; Grade III, *n* = 35), and no separate *a priori* sample-size calculation was performed for these secondary comparisons.

## 3 Results

A total of 76 participants were enrolled, of whom 65 completed follow-up and were included in the final analysis. Eleven participants were excluded due to loss to follow-up (*n* = 6), re-injury (*n* = 3), or surgical intervention due to osteochondral lesions (*n* = 2). The participant flow diagram is presented in Figure 1.

**Figure 1:**
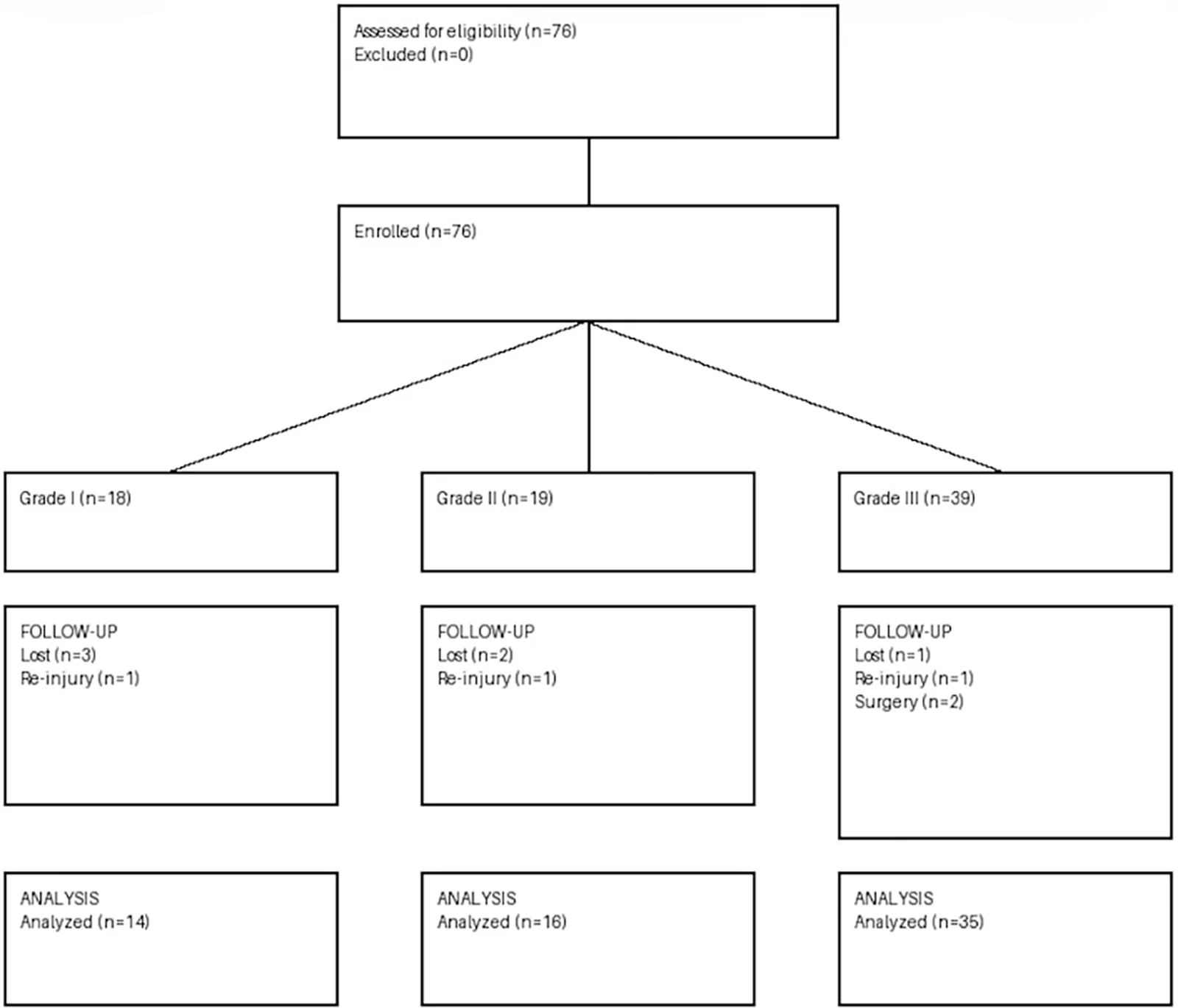
Participant flow diagram according to injury severity classification.

The final sample comprised 65 adolescents [37 males [56.9%] and 28 females [43.1%]] aged 12–17 years. Baseline characteristics are presented in Table 1. No serious adverse events related to the testing procedures were reported.

**Table 1:** Baseline characteristics of participants according to injury severity.

	Grade I (*n* = 14)	Grade II (*n* = 16)	Grade III (*n* = 35)	Test statistic	p
Age in years (median (IQR))	13 (2)	14 (3)	15 (2)	H = 9.101	**0**.**011**
Sex (male/female)	4/10	10/6	23/12	*χ*2 = 5.985	0.052
Height in cm (median (IQR))	166.5 (22)	170.5 (11)	178.0 (17)	H = 7.085	**0**.**029**
Weight in kg (median (IQR))	62.5 (20)	62.0 (20)	71.0 (20)	H = 4.079	0.130
BMI in kg/m^2^ (median (IQR))	20.4 (4)	20.4 (5)	21.5 (3)	H = 1.334	0.513

H, Kruskal–Wallis test statistic; χ^2^, Chi-square test statistic; IQR, interquartile range.

The bold values indicate statistically significant differences (*p* < 0.05).

Significant differences between injury-severity groups were observed for age (*p* = 0.011) and height (*p* = 0.029), whereas no statistically significant differences were observed for sex distribution, body weight, or BMI. Participants in the Grade III group were generally older and taller than those in the lower-severity groups.

### 3.1 Injury severity distribution and initial management

Based on radiologic assessment, 14 patients (21.5%) were classified as Grade I, 16 (24.6%) as Grade II, and 35 (53.8%) as Grade III injuries (Table 2). The distribution of injury severity differed significantly across groups, with the majority of patients classified as Grade III (*χ*^2^ = 12.400, *p* = 0.002).

**Table 2:** Distribution of patients according to injury severity.

Severity	Distribution	Chi-square test statistic	p
Grade I	14 (21.5%)	12.400	**0.002**
Grade II	16 (24.6%)		
Grade III	35 (53.8%)		

The bold values indicate statistically significant differences (*p* < 0.05).

No significant differences in injury severity distribution were observed between the original treatment groups (Table 3). The proportions of Grade I–III injuries were similar between the PRICE and PEACE and LOVE groups.

**Table 3:** Distribution of patients by injury severity in primary treatment groups.

Severity	PRICE (*n* = 32)	PEACE and LOVE (*n* = 33)	Chi-square test statistic	p
Grade I	7 (21.9%)	7 (21.2%)	0.013	0.993
Grade II	8 (25.0%)	8 (24.2%)		
Grade III	17 (53.1%)	18 (54.5%)		

Temporary plaster cast immobilization was applied in 30 patients (46.2%) in the emergency department. When applied, plaster immobilization was discontinued at the first outpatient assessment, which occurred within 1–4 days after the emergency department visit. Thereafter, all participants were provided with a lace-up ankle brace, including those who had not received plaster immobilization. The distribution of plaster immobilization across injury severity groups (Grade I–III) did not differ significantly (*p* = 0.627), indicating that its use was not associated with injury severity in this cohort (Table 4). As the impact of temporary immobilization on functional outcomes was not a primary objective of the present analysis and has been addressed in a previous study, no further analyses were performed.

**Table 4:** Distribution of plaster cast immobilization according to injury severity.

Severity	Cast immobilization applied	No immobilization applied	Chi-square test statistic	*p*
Grade I	6 (20.0%)	8 (22.9%)	0.935	0.627
Grade II	6 (20.0%)	10 (28.6%)		
Grade III	18 (60.0%)	17 (48.6%)		

### 3.2 Functional outcomes according to injury severity

Functional outcomes according to injury severity are summarized in Table 5. Mixed repeated-measures ANOVA revealed significant main effects of time for most strength, ROM, and dynamic balance outcomes, indicating changes in the measured functional outcomes across the follow-up period. Significant main effects of injury severity were observed for ROM at 120°/s and YBT-CS, whereas no significant severity effects were found for the remaining outcomes. No statistically significant Severity × Time interactions were detected for any outcome. The temporal changes in IN and EV strength, ROM, and dynamic balance signed side-to-side differences across injury severity groups are illustrated in Figures 2–5.

**Figure 2:**
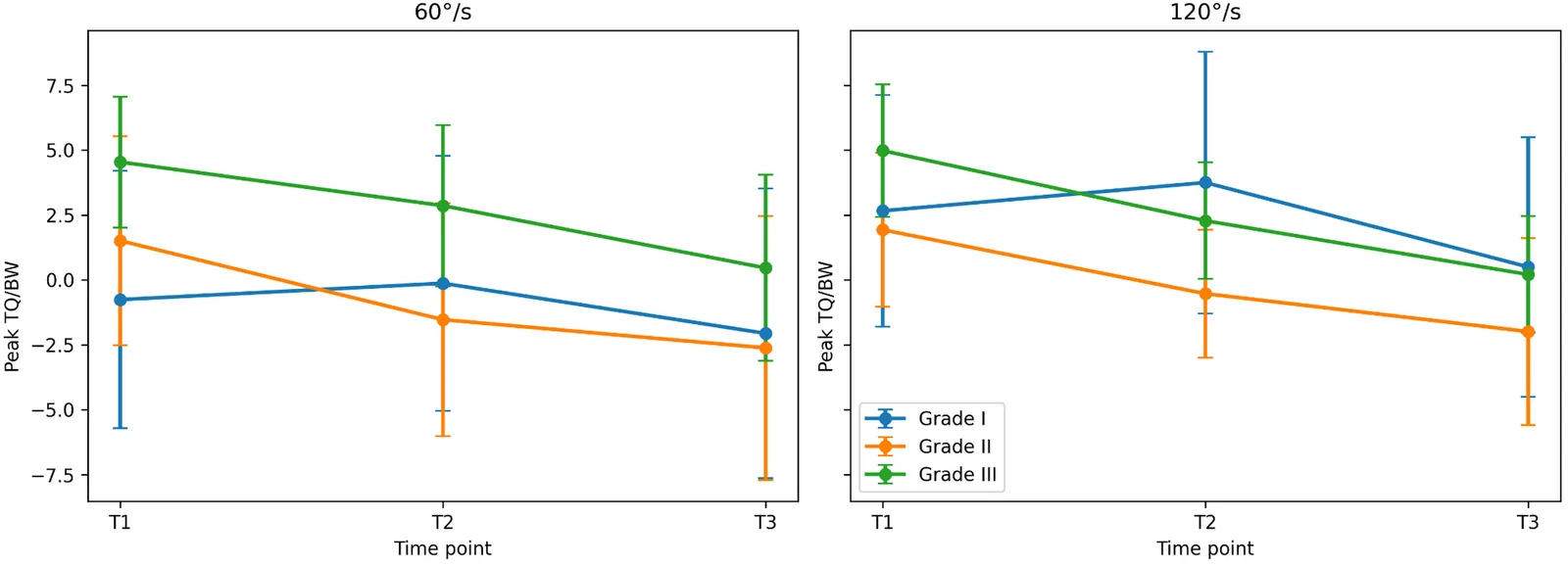
Inversion strength (peak TQ/BW signed side-to-side difference, %) (mean ± 95%, CI).

**Figure 3:**
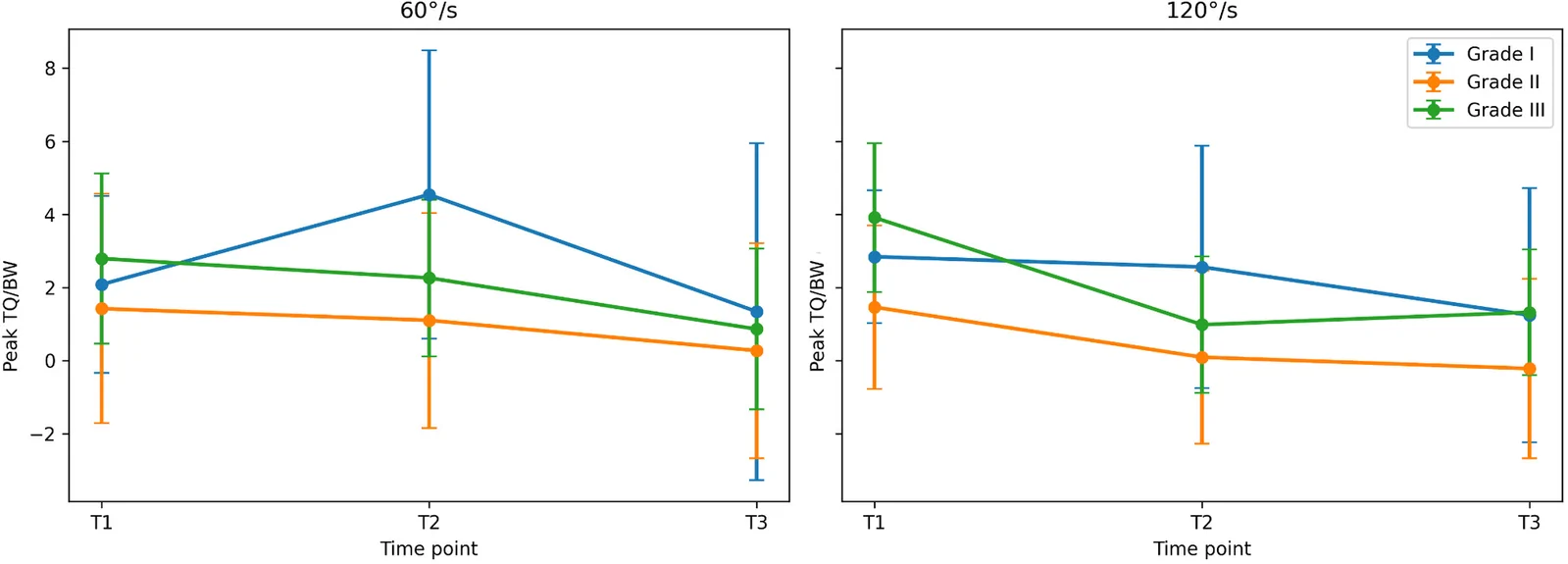
Eversion strength (peak TQ/BW signed side-to-side difference, %) (mean ± 95%, CI).

**Figure 4:**
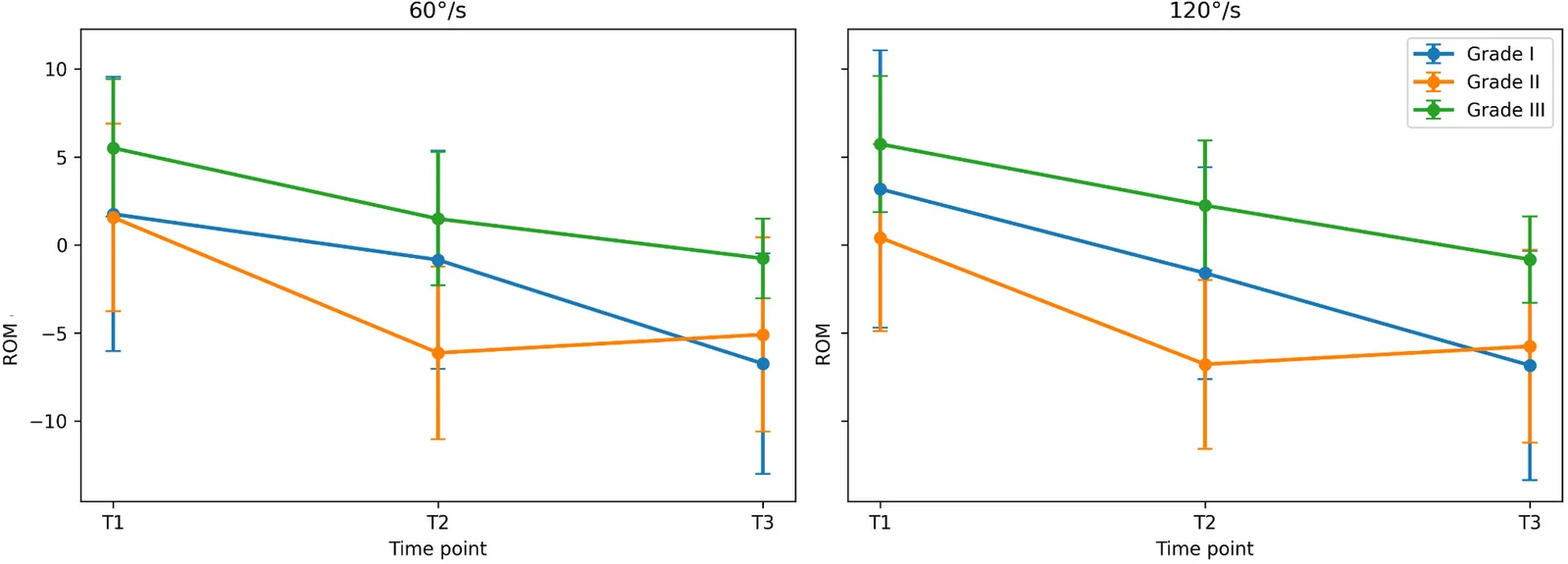
ROM signed side-to-side difference (degrees) (mean ± 95%, CI).

**Figure 5:**
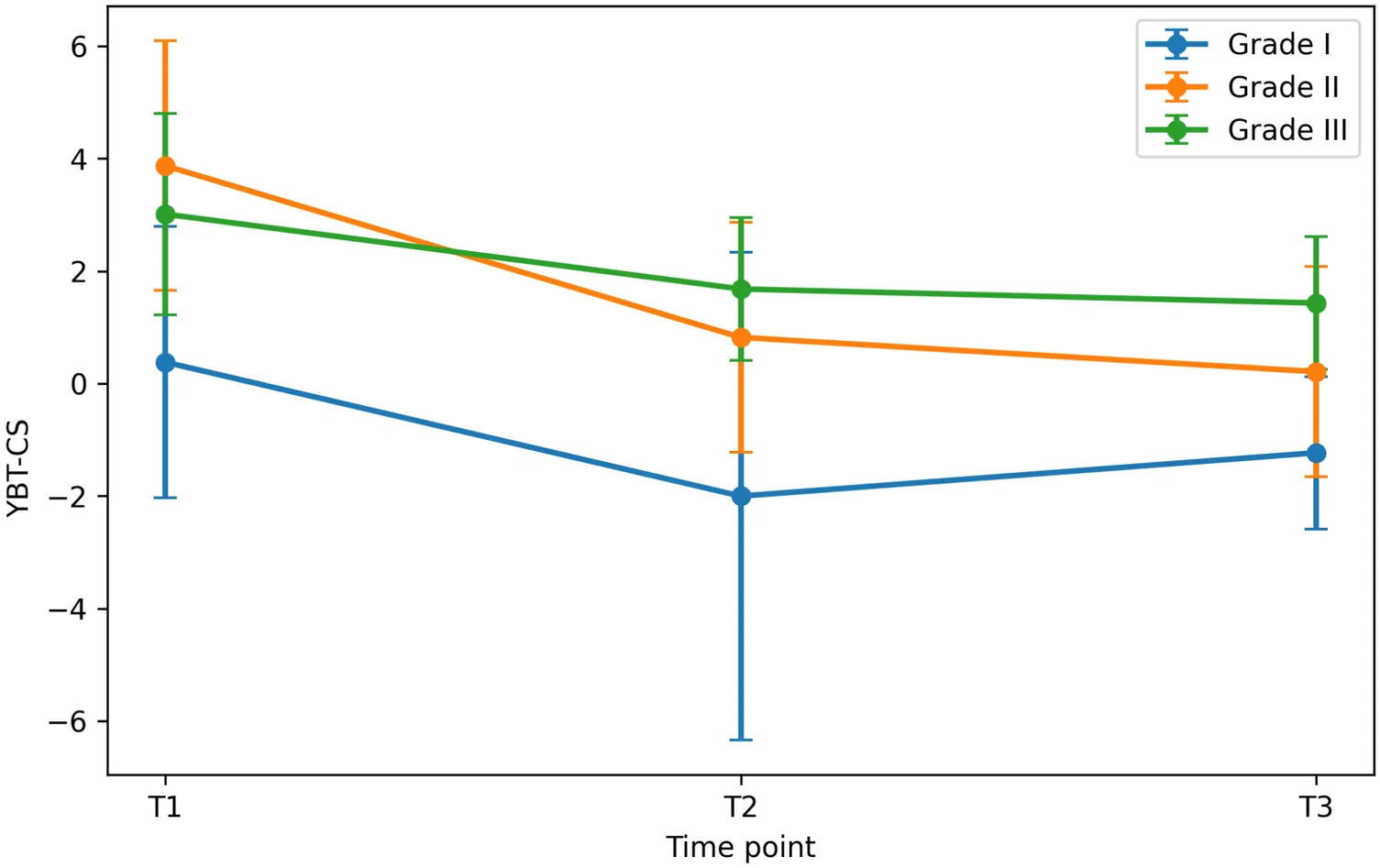
Dynamic balance (YBT-CS signed side-to-side difference, %) (mean ± 5%, CI).

**Table 5:** Functional outcomes according to injury severity over time.

Variable	Time	Severity			Time effect			Severity effect			Time × Severity		
		Grade I	Grade II	Grade III	df	F, *η*p^2^	P	df	F, ηp^2^	p	df	F, ηp^2^	p
Peak TQ/BW IN (U–I) % at 60°/s	T1	−0.76 (8.58)	1.51 (7.55)	4.54 (7.32)									
	T2	−0.13 (8.51)	−1.53 (8.41)	2.86 (9.04)	1.78, 110.07	4.028**,** 0.061	**0**.**025**	2, 62	1.821,0.055	0.170	3.55, 110.07	0.496, 0.016	0.717
	T3	−2.06 (9.66)	−2.62 (9.54)	0.46 (10.42)									
Peak TQ/BW EV (U–I) % at 60°/s	T1	2.08 (4.20)	1.42 (5.89)	2.79 (6.78)									
	T2	4.54 (6.83)	1.10 (5.52)	2.26 (6.24)	2, 124	2.696, 0.042	0.071	2, 62	0.415, 0.013	0.662	4, 124	0.739, 0.023	0.567
	T3	1.34 (7.98)	0.27 (5.53)	0.86 (6.41)									
ROM (U–I)° at 60°/s	T1	1.75 (13.47)	1.56 (9.99)	5.50 (11.35)									
	T2	−0.85 (10.74)	−6.13 (9.19)	1.48 (11.02)	2, 124	11.904, 0.161	**<0**.**001**	2,62	2.831, 0.084	0.067	4, 124	0.854, 0.027	0.494
	T3	−6.74 (10.82)	−5.09 (10.34)	−0.77 (6.58)									
Peak TQ/BW IN (U–I) % at 120°/s	T1	2.66 (7.72)	1.93 (5.57)	4.98 (7.41)									
	T2	3.75 (8.72)	−0.53 (4.63)	2.28 (6.53)	1.61, 98.28	7.315, 0.107	**0**.**002**	2, 61*	1.323, 0.042	0.274	3.22, 98.28	0.808, 0.026	0.500
	T3	0.50 (8.67)	−1.99 (6.75)	0.21 (6.53)									
Peak TQ/BW EV (U–I) % at 120°/s	T1	2.84 (3.14)	1.46 (4.20)	3.91 (5.94)									
	T2	2.56 (5.74)	0.09 (4.44)	0.98 (5.45)	1.73, 106.08	4.391, 0.067	**0**.**019**	2, 61*	0.942, 0.030	0.395	3.48, 106.08	0.756, 0.024	0.539
	T3	1.24 (6.02)	−0.22 (4.61)	1.32 (5.00)									
ROM (U–I)° at 120°/s	T1	3.17 (13.63)	0.41 (9.95)	5.72 (11.22)									
	T2	−1.60 (10.42)	−6.78 (9.00)	2.24 (10.74)	2, 122	12.658, 0.172	**<0**.**001**	2, 61*	3.967, 0.115	**0**.**024**	4, 122	0.776, 0.025	0.543
	T3	−6.84 (11.27)	−5.76 (10.27)	−0.83 (7.13)									
YBT-CS (U–I) %	T1	0.38 (4.17)	3.87 (4.16)	3.01 (5.22)									
	T2	−2.00 (7.50)	0.82 (3.84)	1.68 (3.69)	1.79, 111.08	7.324, 0.106	**0**.**002**	2, 62	4.574, 0.129	**0**.**014**	3.58, 111.08	0.658, 0.021	0.606
	T3	−1.23 (2.35)	0.21 (3.50)	1.43 (3.43)									

*Analyses were conducted per protocol and were based on complete data for each outcome across all three assessment time points. Missing data were not imputed. For the 120°/s outcomes, one participant was excluded from the repeated-measures analyses because data from the first assessment were unavailable due to a technical malfunction of the isokinetic dynamometer; therefore, these analyses included 64 participants.

Values are presented as mean (SD). Outcomes are expressed as signed side-to-side differences, calculated as uninjured−injured. Positive values indicate better performance of the uninjured limb, negative values indicate better performance of the injured limb, and values closer to zero indicate greater interlimb symmetry. Functional performance was assessed at three time points: T1 (1–2 weeks post-injury), T2 (5–7 weeks), and T3 (12–15 weeks). Isokinetic testing was performed at angular velocities of 60°/s and 120°/s for inversion (IN) and eversion (EV) strength and range of motion (ROM). Dynamic balance was assessed using the Y-Balance Test composite score (YBT-CS). Statistical analysis was conducted using mixed repeated-measures analysis of variance (ANOVA), with Time (T1–T3) as the within-subject factor and Severity (Grade I–III) as the between-subject factor. Main effects of Time and Severity, as well as Time × Severity interactions, are reported.

The bold values indicate statistically significant differences (*p* < 0.05).

To further characterize the changes underlying the signed side-to-side differences, injured- and uninjured-limb values were examined separately (Table 6). Descriptively, the limb-specific data were consistent with no systematic deterioration of the uninjured limb across the follow-up period, while injured-limb performance generally improved from T1 to T3. In the additional longitudinal analyses of injured-limb values (Table 7), significant main effects of Time were observed for all measured outcomes (all *p* < 0.001). No statistically significant main effects of Severity were identified for any injured-limb outcome (all *p* > 0.05). Similarly, no statistically significant Severity × Time interactions were identified.

**Table 6:** Injured- and uninjured-limb functional performance across follow-up according to injury severity. Values are presented as mean (SD).

Variable	Time	Grade I injured	uninjured	Grade II injured	uninjured	Grade III injured	uninjured
Peak TQ/BW IN % at 60°/s	T1	23.56 (8.87)	22.80 (9.44)	20.19 (8.45)	21.69 (8.32)	18.24 (8.24)	22.78 (9.35)
	T2	30.60 (9.04)	30.47 (10.71)	25.67 (11.83)	24.14 (9.24)	24.59 (7.78)	27.45 (9.06)
	T3	35.28 (10.36)	33.21 (12.11)	30.46 (13.03)	27. 84 (9.70)	30.00 (12.07)	30.46 (11.85)
Peak TQ/BW EV % at 60°/s	T1	21.35 (6.85)	23.43 (9.48)	22.61 (6.83)	24.03 (8.29)	19.78 (8.34)	22.60 (6.83)
	T2	24.13 (5.34)	28.67 (9.91)	23.69 (7.96)	24.79 (8.78)	23.34 (6.86)	25.60 (7.28)
	T3	28.37 (8.24)	29.71 (8.96)	27.28 (9.66)	27.55 (9.11)	26.81 (8.60)	27.67 (7.91)
ROM° at 60°/s	T1	68.21 (17.56)	69.96 (22.50)	63.15 (10.88)	64.71 (9.91)	67.70 (15.01)	73.21 (12.14)
	T2	75.07 (21.82)	74.22 (20.98)	76.50 (13.73)	70.37 (13.20)	73.62 (15.52)	75.10 (12.40)
	T3	82.35 (13.74)	75.61 (16.27)	79.94 (16.30)	74.86 (17.91)	77.00 (14.46)	76.23 (12.77)
Peak TQ/BW IN % at 120°/s	T1	19.14 (7.69)	21.80 (7.68)	17.17 (6.31)	19.09 (5.56)	17.13 (7.53)	22.11 (6.06)
	T2	24.46 (8.24)	28.21 (10.25)	21.46 (8.99)	20.93 (7.62)	21.82 (7.20)	24.09 (7.15)
	T3	28.34 (8.70)	28.84 (10.54)	24.91 (11.12)	22.92 (7.38)	27.01 (10.21)	27.23 (8.27)
Peak TQ/BW EV % at 120°/s	T1	17.24 (5.75)	20.08 (7.31)	17.84 (6.18)	19.30 (5.47)	15.29 (6.28)	19.20 (5.62)
	T2	19.56 (5.95)	22.13 (7.38)	19.66 (7.35)	19.75 (6.12)	19.13 (5.37)	20.12 (5.61)
	T3	23.07 (7.08)	24.31 (8.29)	22.22 (7.68)	22.00 (6.51)	21.34 (5.99)	22.67 (5.75)
ROM° at 120°/s	T1	66.18 (17.89)	69.35 (22.34)	64.03 (10.84)	64.44 (9.91)	66.93 (14.75)	72.65 (12.17)
	T2	74.97 (22.14)	73.37 (22.32)	76.96 (14.21)	70.18 (13.93)	72.58 (18.25)	74.82 (15.42)
	T3	82.43 (13.63)	75.59 (16.15)	80.69 (16.23)	74.93 (18.06)	77.40 (14.11)	76.57 (12.40)
YBT-CS %	T1	91.46 (9.94)	91.84 (8.88)	88.37 (6.70)	92.24 (7.09)	88.58 (17.97)	91.59 (17.98)
	T2	98.19 (9.74)	96.19 (12.40)	96.24 (9.24)	97.06 (7.61)	93.37 (6.74)	95.05 (6.77)
	T3	101.75 (13.02)	100.52 (13.92)	99.35 (8.26)	99.56 (7.26)	95.74 (6.56)	97.17 (6.04)

**Table 7:** Absolute injured-limb functional outcomes according to injury severity over time.

Variable	Time	Severity			Time effect			Severity effect			Time × Severity		
		Grade I	Grade II	Grade III	df	F, ηp^2^	P	df	F, ηp^2^	p	df	F, ηp^2^	p
Peak TQ/BW IN % at 60°/s	T1	23.56 (8.87)	20.19 (8.45)	18.24 (8.24)									
	T2	30.60 (9.04)	25.67 (11.83)	24.59 (7.78)	1.49, 92.34	48.79, 0.440	**<0**.**001**	2, 62	2.04, 0.062	0.138	2, 98	0.12, 0.004	0.948
	T3	35.28 (10.36)	30.46 (13.03)	30.00 (12.07)									
Peak TQ/BW EV % at 60°/s	T1	21.35 (6.85)	22.61 (6.83)	19.78 (8.34)									
	T2	24.13 (5.34)	23.69 (7.96)	23.34 (6.86)	2, 124	32.78, 0.346	**<0**.**001**	2, 62	0.26, 0.008	0.775	4, 124	0.70, 0.022	0.593
	T3	28.37 (8.24)	27.28 (9.66)	26.81 (8.59)									
ROM° at 60°/s	T1	68.21 (17.56)	63.15 (10.88)	67.70 (15.01)									
	T2	75.07 (21.82)	76.50 (13.73)	73.62 (15.52)	2, 124	28.94, 0.318	**<0**.**001**	2, 62	0.17, 0.005	0.845	4, 124	1.39, 0.043	0.241
	T3	82.35 (13.74)	79.94 (16.30)	77.00 (14.46)									
Peak TQ/BW IN % at 120°/s	T1	19.14 (7.69)	17.17 (6.31)	17.13 (7.53)									
	T2	24.46 (8.24)	21.46 (8.99)	21.82 (7.20)	1.49, 90.75	36.92, 0.377	**<0**.**001**	2, 61*	0.59, 0.019	0.556	2.98, 90.75	0.27, 0.009	0.845
	T3	28.34 (8.70)	24.91 (11.12)	27.01 (10.21)									
Peak TQ/BW EV % at 120°/s	T1	17.24 (5.75)	17.84 (6.18)	15.29 (6.28)									
	T2	19.56 (5.95)	19.66 (7.35)	19.13 (5.37)	2, 122	38.51, 0.387	**<0**.**001**	2, 61*	0.44, 0.014	0.645	4, 122	0.81, 0.026	0.522
	T3	23.07 (7.08)	22.22 (7.68)	21.34 (5.99)									
ROM° at 120°/s	T1	66.18 (17.89)	64.03 (10.84)	66.93 (14.75)									
	T2	74.97 (22.14)	76.96 (14.21)	72.58 (18.25)	2, 122	28,00, 0.315	**<0**.**001**	2, 61*	0.16, 0.005	0.850	4, 122	1.01, 0.032	0.408
	T3	82.43 (13.63)	80.69 (16.23)	77.40 (14.11)									
YBT-CS %	T1	91.46 (9.94)	88.37 (6.70)	88.58 (17.97)									
	T2	98.19 (9.74)	96.24 (9.24)	93.37 (6.74)	1.23, 75.96	19.56, 0.240	**<0**.**001**	2, 62	1.50, 0.046	0.232	2.45, 75.96	0.42, 0.013	0.702
	T3	101.75 (13.02)	99.35 (8.26)	95.74 (6.56)									

*Analyses were conducted per protocol and were based on complete data for each outcome across all three assessment time points. Missing data were not imputed. For the 120°/s outcomes, one participant was excluded from the repeated-measures analyses because data from the first assessment were unavailable due to a technical malfunction of the isokinetic dynamometer; therefore, these analyses included 64 participants.

Values are presented as mean (SD). Outcomes are expressed as absolute values measured for the injured limb rather than signed side-to-side differences. Thus, changes over time represent changes in the performance of the injured limb itself and are not influenced by concurrent changes in the uninjured limb. Functional performance was assessed at three time points: T1 (1–2 weeks post-injury), T2 (5–7 weeks), and T3 (12–15 weeks). Isokinetic testing was performed at angular velocities of 60°/s and 120°/s for inversion (IN) and eversion (EV) strength and range of motion (ROM). Dynamic balance was assessed using the Y-Balance Test composite score (YBT-CS). Statistical analysis was conducted using mixed repeated-measures analysis of variance (ANOVA), with Time (T1–T3) as the within-subject factor and Severity (Grade I–III) as the between-subject factor. Main effects of Time and Severity, as well as Time × Severity interactions, are reported.

The bold values indicate statistically significant differences (*p* < 0.05).

In the covariate-adjusted linear mixed-effects models of the primary signed side-to-side difference outcomes (Table 8), no statistically significant Severity × Time interactions were identified for any of the seven outcomes (all *p* > 0.05). Significant Time effects were observed for PEAK TQ/BW IN and EV at 120°/s, ROM at 60°/s and 120°/s, and YBT-CS, whereas the Time effects for PEAK TQ/BW IN and EV at 60°/s were not statistically significant. A significant Severity main effect was identified only for YBT-CS (*p* = 0.045); the Severity effect for ROM at 120°/s observed in the repeated-measures ANOVA was not statistically significant in the adjusted model (*p* = 0.090). Neither age nor original treatment allocation was significantly associated with any of the signed side-to-side difference outcomes. Detailed fixed-effect estimates with 95% confidence intervals are provided in Supplementary Tables S1-S2.

**Table 8:** Fixed effects from covariate-adjusted linear mixed-effects models of signed side-to-side differences (U−I) in functional outcomes.

Outcome	Severity	Time	Severity × Time	Age	Treatment
PEAK TQ/BW IN 60°/s	F(2, 60.84) = 1.08; *p* = 0.348	F(2, 62.01) = 3.03; *p* = 0.056	F(4, 62.01) = 0.51; *p* = 0.732	F(1, 60.02) = 1.32; *p* = 0.254	F(1, 60.02) = 0.05; *p* = 0.828
PEAK TQ/BW EV 60°/s	F(2, 60.08) = 0.43; *p* = 0.650	F(2, 62.00) = 2.45; *p* = 0.095	F(4, 62.00) = 0.76; *p* = 0.555	F(1, 60.00) = 0.03; *p* = 0.854	F(1, 60.00) = 2.29; *p* = 0.135
PEAK TQ/BW IN 120°/s	F(2, 60.35) = 1.24; *p* = 0.297	**F(2, 62.01)** **=** **6.35; *p*** **=** **0.003**	F(4, 62.06) = 0.93; *p* = 0.454	F(1, 61.05) = 0.41; *p* = 0.524	F(1, 60.20) = 0.05; *p* = 0.818
PEAK TQ/BW EV 120°/s	F(2, 60.41) = 1.00; *p* = 0.375	**F(2, 61.93)** **=** **3.81; *p*** **=** **0.027**	F(4, 61.97) = 0.68; *p* = 0.608	F(1, 61.11) = 0.16; *p* = 0.691	F(1, 60.18) = 0.04; *p* = 0.836
ROM 60°/s	F(2, 60.55) = 2.02; *p* = 0.142	**F(2, 62.03)** **=** **9.94; *p*=<0.001**	F(4, 62.03) = 1.05; *p* = 0.388	F(1, 60.07) = 1.51; *p* = 0.224	F(1, 60.07) = 1.05; *p* = 0.309
ROM 120°/s	F(2, 60.28) = 2.51; *p* = 0.090	**F(2, 58.67)** **=** **10.24; *p*=<0.001**	F(4, 58.71) = 0.75; *p* = 0.560	F(1, 60.44) = 1.97; *p* = 0.165	F(1, 60.02) = 1.38; *p* = 0.245
YBT-CS	**F(2, 61.05)** **=** **3.27; *p*** **=** **0.045**	**F(2, 62.01)** **=** **7.33; *p*** **=** **0.001**	F(4, 62.01) = 0.76; *p* = 0.555	F(1, 60.03) = 0.84; *p* = 0.362	F(1, 60.03) = 1.61; *p* = 0.210

Values are presented as *F* (numerator df, denominator df) and *p*-values. Signed side-to-side differences were calculated as uninjured minus injured limb (U−I). Positive values indicate higher values for the uninjured limb, negative values indicate higher values for the injured limb, and values closer to zero indicate greater interlimb symmetry. Severity, Time, Severity × Time, age, and original treatment allocation (PRICE vs PEACE and LOVE) were included as fixed effects, with participant included as a random effect. The Severity × Time interaction was the primary effect of interest. Statistically significant results (*p* < 0.05) are shown in bold. Detailed fixed-effect estimates with 95% confidence intervals are provided in Supplementary Tables S1-S2.

In covariate-adjusted linear mixed-model sensitivity analyses of injured-limb values, no statistically significant Severity × Time interactions were identified for any of the seven outcomes (all *p* > 0.05). No significant Severity main effects were observed. Significant Time effects were identified for all outcomes (all *p* < 0.001). Age was significantly associated with ROM at 60°/s (*p* = 0.002) and ROM at 120°/s (*p* = 0.004), whereas original treatment allocation was not significantly associated with any outcome (all *p* > 0.05). A broader sensitivity analysis additionally adjusting for BMI produced the same overall pattern, with no statistically significant Severity or Severity × Time effects. Full model results, including fixed-effect estimates with 95% confidence intervals, are provided in Supplementary Tables S3–S5.

## 4 Discussion

### 4.1 Key results of the study

The present exploratory *post hoc* secondary analysis examined whether structural injury severity was associated with the short-term recovery of objective functional measures following adolescent LAS. The data were originally collected within a RCT designed to compare PRICE and PEACE and LOVE rehabilitation strategies; the present severity-based analysis was not prespecified in the original trial protocol. Participants were therefore analyzed observationally according to Grade I–III injury severity. No statistically significant Severity × Time interactions were detected for the measured strength, ROM, or dynamic balance outcomes.

Significant main effects of injury severity were observed for selected outcomes, including ROM at 120°/s and YBT-CS. These findings suggest that structural injury severity may be associated with the overall level of impairment in selected objective measures; however, the present secondary analysis cannot establish an independent or causal effect of injury grade on recovery.

The covariate-adjusted LMM analysis of the signed side-to-side differences supported the primary finding, with no statistically significant Severity × Time interactions identified for any outcome. Notably, the Severity main effect for ROM at 120°/s observed in the repeated-measures ANOVA was not retained after adjustment for age and treatment allocation, whereas the Severity main effect for YBT-CS remained statistically significant. These findings suggest that the ROM at 120°/s severity-related difference was less robust to covariate adjustment, while the overall severity-related difference in YBT-CS was more consistent across analytical approaches.

### 4.2 Age-related differences in injury severity

Age and height differed significantly across the injury-severity groups in the present cohort, with younger and shorter adolescents more commonly represented in the Grade I group and older, taller participants more commonly represented in the Grade III group. These baseline differences should be interpreted cautiously because age, body size, and physical maturation may themselves influence muscle strength, dynamic balance, neuromuscular control, and recovery independently of structural injury grade. These findings may generate the hypothesis that age-related differences in injury severity are partly influenced by factors associated with physical maturation. One possible explanation is that younger children may present to emergency departments after relatively mild injuries; however, this was not directly evaluated in the present study. Pediatric pain assessment can also be influenced by age-dependent differences in the ability to verbalize and quantify pain, with younger children relying more on behavioral and indirect indicators than on self-report (21).

These findings could be influenced not only by physical maturation but also by differences in the type and intensity of sports participation between younger and older adolescents (9). Older adolescents are generally involved in higher-intensity and more frequent sports participation, which exposes them to greater mechanical loads and may increase the chance of more severe ligament injuries (22).

In addition, older adolescents are often exposed to greater biomechanical loads due to increased body mass, movement velocity, as well as participation in competitive sports (1, 9). Moreover, they may be more likely to continue activity despite pain, which could contribute to more severe injuries.

Adolescent developmental changes may also contribute to injury risk, as maturation-related factors can influence neuromuscular control and tissue tolerance, potentially increasing the incidence of more severe ankle sprains in older adolescents. This concept is partially supported by a previous study reporting an association between timing of menarche and first ankle sprain in adolescent female soccer players; however, that study was conducted exclusively in female athletes and may not be fully generalizable to the broader adolescent population (23). Therefore, age-related differences in injury severity should be considered when interpreting rehabilitation outcomes, as injury grade may partially reflect differences in exposure, maturation, and healthcare-seeking behavior rather than structural damage alone. One possible explanation for the relatively high proportion of Grade III injuries in the present cohort is that adolescents with milder sprains and limited pain or functional impairment may be less likely to present to the emergency department or participate in a clinical study. This potential selection effect may have contributed to the overrepresentation of more severe injuries in the present cohort and should be considered when generalizing the findings to broader community populations.

### 4.3 Comparison with previous literature

The findings of this exploratory analysis partially align with previous studies suggesting that structural injury severity alone may not fully explain subsequent functional outcomes (2, 5). Some authors suggest that even in cases of more severe ligament tears, functional recovery may be comparable to that seen in minor injuries if appropriate rehabilitation is provided (10). In contrast to some traditional views, our findings suggest that structural classification alone may not fully explain short-term changes in the objective functional measures assessed in this study. Taken together, the present observations suggest that recovery of the selected objective functional measures may not be determined solely by initial ligament injury grade.

### 4.4 Role of structural and functional assessment in ankle sprain management

When assessing acute ankle sprains and the completion of healing, it is important to consider not only the grades of ligament injury, which remain useful for the initial description of the injury, but also functional factors (1, 24, 25), such as neuromuscular and proprioceptive disorders, which are closely related to functional instability and rehabilitation outcomes (10).

Current literature suggests that the degree of ligament injury does not always correspond directly to functional status or patient-perceived recovery. It is believed that these post-traumatic changes in the sensorimotor system have a greater impact on function than the structural injury itself. Therefore, a structural diagnosis alone does not suitably reflect the patient's functional status and may limit the selection of an optimal rehabilitation strategy. This approach allows for a more accurate assessment of the patient's functional status and justifies the application of treatment, reducing the need for excessive immobilization in patients who retain good functional capacity. Most lateral ankle ligament injuries can be successfully treated conservatively, and surgical treatment is rarely necessary and is most often used only in cases of persistent mechanical instability or unsuccessful rehabilitation (9, 26).

This concept is also supported by previous studies using patient-reported outcome measures such as the Foot and Ankle Ability Measure (FAAM) and Cumberland Ankle Instability Tool (CAIT), which have shown that greater anatomical injury severity does not necessarily correspond to greater perceived disability or poorer functional recovery (27). These findings suggest a similar pattern to that observed in the present study, namely that structural injury grade alone may not fully explain the course of recovery. The FAAM and CAIT were not used in the present study, but they represent complementary tools that may help capture patient-perceived aspects of recovery not reflected by objective biomechanical measures (28).

### 4.5 Functional impairments and neuromuscular consequences

Patients with ankle injuries often exhibit not only localized problems in the ankle but also impairments in muscle strength and motor control throughout the entire lower limb (29). Even severe pain may further negatively affect motor function and movement quality. These functional impairments may lead to abnormal muscle activation and reduced coordination during dynamic tasks, indicating sensorimotor impairments that significantly influence long-term functional status. Persistent impairments in proprioception and neuromuscular control increase the risk of recurrent injuries, leading to functional instability, defined as CAI (30). From a clinical perspective, the present findings suggest that structural injury severity should not necessarily be considered in isolation when evaluating rehabilitation progress. Objective functional measures, including strength and dynamic balance, may provide complementary information alongside structural assessment. However, because return to sport was not evaluated in this study, the present findings do not establish specific return-to-sport criteria.

### 4.6 Functional differences associated with injury severity

Significant main effects of injury severity were observed for ROM at 120°/s and YBT-CS. For ROM at 120°/s, however, the pattern of signed side-to-side differences varied across assessment time points. Grade III injuries showed the largest positive side-to-side difference at T1, whereas by T3 the Grade III mean was closest to zero. Therefore, the severity main effect should not be interpreted as indicating a persistently less favorable ROM pattern in higher-grade injuries. The additional limb-specific analyses provide further context. Although Severity main effects were observed for the signed side-to-side differences in ROM at 120°/s and YBT-CS, no significant Severity effects were identified for injured-limb values. These findings are consistent with the interpretation that the observed severity-related differences in signed side-to-side values reflect differences in interlimb relationships rather than statistically detectable differences in injured-limb performance itself. These differences may partly reflect greater initial mechanical and sensorimotor disruption associated with more severe ligament injury, including complete rupture and avulsion-type injuries. Reduced ROM after severe LAS has previously been associated with pain, swelling, joint effusion, and protective muscle inhibition, all of which may restrict ankle mobility during the early rehabilitation phase (17, 20). Similarly, impairments in dynamic balance may reflect altered proprioceptive input and neuromuscular control resulting from damage to mechanoreceptors within the lateral ligament complex (10, 30).

### 4.7 Study limitations

This study has several limitations. First, the present study was an exploratory *post hoc* secondary analysis of data from a RCT that was originally designed to compare PRICE and PEACE and LOVE rehabilitation strategies. The severity-based research question and analyses were not prespecified in the original trial protocol, and the study was not prospectively designed or powered specifically to compare recovery trajectories among Grade I–III injury groups. Furthermore, the severity groups were unequal in size, with relatively small Grade I (*n* = 14) and Grade II (*n* = 16) subgroups compared with Grade III (*n* = 35). These relatively small and unequal subgroups may have limited statistical power to detect clinically meaningful between-group differences or small-to-moderate Severity × Time interaction effects. Consequently, nonsignificant interactions may reflect limited statistical power and should not be interpreted as evidence of equivalent recovery trajectories. The present findings should therefore be regarded as hypothesis-generating rather than confirmatory. In addition, because seven separate outcome models were examined without adjustment for multiple comparisons, the nominally significant severity effects for ROM at 120°/s and YBT-CS may be susceptible to type I error and should therefore be interpreted cautiously. Second, significant baseline differences in age and height were present between the injury-severity groups. Both age and body size may influence muscle strength, dynamic balance, neuromuscular control, physical maturation, and recovery during adolescence independently of structural injury severity. Covariate-adjusted linear mixed-model sensitivity analyses of injured-limb outcomes incorporating age and original treatment allocation, as well as broader models additionally adjusting for BMI, did not identify statistically significant Severity or Severity × Time effects. These analyses were exploratory and were used to evaluate the robustness of the injured-limb findings to covariate adjustment. Because participants were analyzed observationally according to injury severity, the advantages of randomization in the original treatment comparison do not apply to the Grade I–III severity comparisons. In addition, although temporary plaster immobilization was limited to the acute post-injury period and discontinued at the first outpatient visit, its exact individual duration was not incorporated into the present analysis. Therefore, a potential influence of short-term initial immobilization on early strength, ROM, and dynamic balance outcomes cannot be completely excluded.

A further limitation is that the Grade III category was structurally heterogeneous, encompassing complete ATFL rupture with variable involvement of the CFL and/or PTFL as well as avulsion-type injuries. Although these injuries met the predefined criteria for Grade III LAS, differences in the extent and pattern of ligamentous damage within this group may have contributed to variability in functional outcomes and may limit interpretation of Grade III as a homogeneous injury category. The relatively high proportion of Grade III injuries may also reflect selection characteristics of the emergency and tertiary-care setting, potentially limiting generalizability to community populations in which milder ankle sprains may be more prevalent. In addition, formal inter-rater or intra-rater reliability of the Grade I–III injury-severity classification was not assessed. Therefore, some degree of severity misclassification cannot be excluded, which may have reduced the ability to detect differences between severity groups.

The study used the contralateral uninjured limb as a within-participant comparison rather than a true preinjury reference. In addition, outcomes were expressed as signed side-to-side differences; therefore, negative values indicate greater performance of the injured limb rather than necessarily better recovery. Changes in these values should consequently be interpreted in relation to zero and interlimb symmetry rather than according to numerical direction alone. Physical activity levels during the follow-up period were not systematically monitored. Therefore, potential changes in physical activity or training exposure that may have influenced performance of either limb could not be accounted for.

Another limitation is that the present study assessed recovery using selected objective biomechanical measures rather than a comprehensive evaluation of clinical recovery. Patient-reported outcomes, including pain, perceived instability, self-reported function, psychological readiness, and return-to-sport confidence, were not assessed. Consequently, the measured strength, ROM, and dynamic balance outcomes represent only selected dimensions of recovery and should not be interpreted as a comprehensive assessment of clinical recovery. In addition, although ankle ROM was assessed using isokinetic dynamometry, weight-bearing dorsiflexion (DF) ROM was not specifically evaluated. Isokinetic assessment does not fully capture functional DF mobility under weight-bearing conditions, which may be more relevant during dynamic tasks and landing activities. Previous studies have demonstrated associations between weight-bearing DF ROM and YBT performance, particularly anterior reach distance in athletic populations (31, 32). Therefore, the absence of a specific weight-bearing DF assessment may have influenced interpretation of the dynamic balance findings, and future studies should incorporate standardized functional DF measurements to better clarify their relationship with post-injury recovery.

The per-protocol analysis excluded participants who did not complete follow-up, including three participants who experienced reinjury and two who subsequently required surgery for osteochondral lesions. Because these participants had less favorable or more complicated clinical courses, their exclusion may have biased the analyzed cohort toward better short-term outcomes.

Finally, the follow-up period was limited to 12–15 weeks and therefore reflects only short-term recovery of the measured objective functional outcomes. The study was not designed to evaluate recurrent ankle sprains, development of CAI, long-term return to sport, or longer-term functional outcomes. Therefore, the present findings cannot be extrapolated to longer-term outcomes across injury-severity groups.

## 5 Conclusions

This exploratory *post hoc* secondary analysis found no statistically significant evidence of different short-term recovery trajectories across Grade I–III injury-severity groups for the measured objective outcomes of muscle strength, ROM, and dynamic balance, including in covariate-adjusted LMM analyses. Significant severity-group differences in overall signed side-to-side values were observed for ROM at 120°/s and YBT-CS in the repeated-measures ANOVA; however, after adjustment for age and treatment allocation, the Severity effect remained significant for YBT-CS but not for ROM at 120°/s. No significant Severity effects were identified when injured-limb values were analyzed separately. Prospective studies specifically designed and adequately powered to evaluate injury-severity-related recovery are required to confirm these observations and determine their relevance to longer-term outcomes such as recurrent sprain, return to sport, and CAI.

## Data Availability

The datasets generated and analyzed during the current study are not publicly available due to privacy reasons, but they are available from the corresponding author on reasonable request.
