# Supplementary material for: Association between injury severity and recovery of objective functional measures following adolescent lateral ankle sprain: a secondary cohort analysis
Source: Front Sports Act Living. 2026 Oct 8;8:1966025. doi: 10.3389/fspor.2026.1966025 (PMC13687335; doi:10.3389/fspor.2026.1966025)
Supplement: Supplementary file 1 [file Table1.docx]

Supplementary Material (tables)

## 1.1 Supplementary Tables

Covariate-adjusted linear mixed-effects analyses of the primary signed side-to-side differences (U−I) are presented in Supplementary Tables S1–S2. Models included injury Severity, Time, Severity × Time, age, and original treatment allocation (PRICE vs PEACE and LOVE) as fixed effects, with participant included as a random effect. Omnibus fixed-effect tests are presented in Supplementary Table S1, and corresponding parameter estimates with 95% confidence intervals are presented in Supplementary Table S2. Additional sensitivity analyses of injured-limb functional outcomes are presented in Supplementary Tables S3–S5. The primary sensitivity models included injury Severity, Time, Severity × Time, age, and original treatment allocation as fixed effects. A broader sensitivity analysis additionally included BMI.

**Supplementary Table S1.** Omnibus tests of fixed effects from covariate-adjusted linear mixed-effects models of signed side-to-side differences (U−I) in functional outcomes. Signed side-to-side differences were calculated as uninjured minus injured limb (U−I). Models included Severity, Time, Severity × Time, age, and original treatment allocation (PRICE vs PEACE and LOVE) as fixed effects, with participant included as a random effect. The Severity × Time interaction was the primary effect of interest. *F*, F-statistic; df, degrees of freedom; PEAK TQ/BW, peak torque normalized to body weight; IN, inversion; EV, eversion; ROM, range of motion; YBT-CS, Y-Balance Test composite score. Statistical significance was set at *p* < 0.05.

| **Outcome** | **Fixed effect** | **Numerator df** | **Denominator df** | **F** | **P** |
| --- | --- | --- | --- | --- | --- |
| PEAK TQ/BW IN 60°/s | Severity | 2 | 60.84 | 1.08 | 0.348 |
| PEAK TQ/BW IN 60°/s | Time | 2 | 62.01 | 3.03 | 0.056 |
| PEAK TQ/BW IN 60°/s | Severity × Time | 4 | 62.01 | 0.51 | 0.732 |
| PEAK TQ/BW IN 60°/s | Treatment | 1 | 60.02 | 0.05 | 0.828 |
| PEAK TQ/BW IN 60°/s | Age | 1 | 60.02 | 1.32 | 0.254 |
| PEAK TQ/BW EV 60°/s | Severity | 2 | 60.08 | 0.43 | 0.650 |
| PEAK TQ/BW EV 60°/s | Time | 2 | 62 | 2.45 | 0.095 |
| PEAK TQ/BW EV 60°/s | Severity × Time | 4 | 62 | 0.76 | 0.555 |
| PEAK TQ/BW EV 60°/s | Treatment | 1 | 60 | 2.29 | 0.135 |
| PEAK TQ/BW EV 60°/s | Age | 1 | 60 | 0.03 | 0.854 |
| PEAK TQ/BW IN 120°/s | Severity | 2 | 60.35 | 1.24 | 0.297 |
| PEAK TQ/BW IN 120°/s | Time | 2 | 62.01 | 6.35 | 0.003 |
| PEAK TQ/BW IN 120°/s | Severity × Time | 4 | 62.06 | 0.93 | 0.454 |
| PEAK TQ/BW IN 120°/s | Treatment | 1 | 60.2 | 0.05 | 0.818 |
| PEAK TQ/BW IN 120°/s | Age | 1 | 61.05 | 0.41 | 0.524 |
| PEAK TQ/BW EV 120°/s | Severity | 2 | 60.41 | 1 | 0.375 |
| PEAK TQ/BW EV 120°/s | Time | 2 | 61.93 | 3.81 | 0.027 |
| PEAK TQ/BW EV 120°/s | Severity × Time | 4 | 61.97 | 0.68 | 0.608 |
| PEAK TQ/BW EV 120°/s | Treatment | 1 | 60.18 | 0.04 | 0.836 |
| PEAK TQ/BW EV 120°/s | Age | 1 | 61.11 | 0.16 | 0.691 |
| ROM 60°/s | Severity | 2 | 60.55 | 2.02 | 0.142 |
| ROM 60°/s | Time | 2 | 62.03 | 9.94 | <0.001 |
| ROM 60°/s | Severity × Time | 4 | 62.03 | 1.05 | 0.388 |
| ROM 60°/s | Treatment | 1 | 60.07 | 1.05 | 0.309 |
| ROM 60°/s | Age | 1 | 60.07 | 1.51 | 0.224 |
| ROM 120°/s | Severity | 2 | 60.28 | 2.51 | 0.090 |
| ROM 120°/s | Time | 2 | 58.67 | 10.24 | <0.001 |
| ROM 120°/s | Severity × Time | 4 | 58.71 | 0.75 | 0.560 |
| ROM 120°/s | Treatment | 1 | 60.02 | 1.38 | 0.245 |
| ROM 120°/s | Age | 1 | 60.44 | 1.97 | 0.165 |
| YBT-CS | Severity | 2 | 61.05 | 3.27 | 0.045 |
| YBT-CS | Time | 2 | 62.01 | 7.33 | 0.001 |
| YBT-CS | Severity × Time | 4 | 62.01 | 0.76 | 0.555 |
| YBT-CS | Treatment | 1 | 60.03 | 1.61 | 0.210 |
| YBT-CS | Age | 1 | 60.03 | 0.84 | 0.362 |

**Supplementary Table S2.** Fixed-effect parameter estimates from covariate-adjusted linear mixed-effects models of signed side-to-side differences (U−I) in functional outcomes. Reference categories were Grade III for Severity, T3 for Time, and PEACE and LOVE for original treatment allocation. Coefficients for Severity and Time are conditional on the reference categories because the models included Severity × Time interactions. Interaction coefficients represent departures from the corresponding additive main effects. U−I, uninjured minus injured limb; SE, standard error; CI, confidence interval; PEAK TQ/BW, peak torque normalized to body weight; IN, inversion; EV, eversion; ROM, range of motion; YBT-CS, Y-Balance Test composite score; PRICE, Protection, Rest, Ice, Compression, Elevation. Statistical significance was set at *p* < 0.05.

| **Outcome** | **Parameter** | **Estimate** | **SE** | **df** | **t** | **P** | **95% CI** |
| --- | --- | --- | --- | --- | --- | --- | --- |
| PEAK TQ/BW IN 60°/s | Intercept | -9.81 | 9.05 | 62.09 | -1.08 | 0.283 | -27.89 to 8.28 |
| PEAK TQ/BW IN 60°/s | Grade I vs Grade III | -1.49 | 3.29 | 65.58 | -0.45 | 0.652 | -8.07 to 5.08 |
| PEAK TQ/BW IN 60°/s | Grade II vs Grade III | -2.64 | 3.05 | 62.14 | -0.87 | 0.389 | -8.73 to 3.45 |
| PEAK TQ/BW IN 60°/s | T1 vs T3 | 4.07 | 1.64 | 62.02 | 2.49 | 0.016 | 0.80 to 7.35 |
| PEAK TQ/BW IN 60°/s | T2 vs T3 | 2.39 | 1.25 | 62 | 1.91 | 0.061 | -0.11 to 4.90 |
| PEAK TQ/BW IN 60°/s | T1 × Grade I interaction | -2.77 | 3.07 | 62.02 | -0.91 | 0.369 | -8.90 to 3.35 |
| PEAK TQ/BW IN 60°/s | T2 × Grade I interaction | -0.46 | 2.34 | 62 | -0.2 | 0.846 | -5.14 to 4.23 |
| PEAK TQ/BW IN 60°/s | T1 × Grade II interaction | 0.05 | 2.93 | 62.02 | 0.02 | 0.986 | -5.80 to 5.90 |
| PEAK TQ/BW IN 60°/s | T2 × Grade II interaction | -1.3 | 2.24 | 62 | -0.58 | 0.563 | -5.77 to 3.17 |
| PEAK TQ/BW IN 60°/s | PRICE vs PEACE and LOVE | 0.39 | 1.78 | 60.02 | 0.22 | 0.828 | -3.17 to 3.94 |
| PEAK TQ/BW IN 60°/s | Age | 0.67 | 0.59 | 60.02 | 1.15 | 0.254 | -0.50 to 1.84 |
| PEAK TQ/BW EV 60°/s | Intercept | -1.3 | 6.64 | 61.24 | -0.2 | 0.846 | -14.58 to 11.98 |
| PEAK TQ/BW EV 60°/s | Grade I vs Grade III | 0.57 | 2.19 | 64.95 | 0.26 | 0.795 | -3.80 to 4.94 |
| PEAK TQ/BW EV 60°/s | Grade II vs Grade III | -0.57 | 2.01 | 61.96 | -0.28 | 0.779 | -4.59 to 3.45 |
| PEAK TQ/BW EV 60°/s | T1 vs T3 | 1.93 | 0.98 | 62 | 1.96 | 0.054 | -0.03 to 3.89 |
| PEAK TQ/BW EV 60°/s | T2 vs T3 | 1.4 | 1.06 | 62 | 1.32 | 0.193 | -0.73 to 3.53 |
| PEAK TQ/BW EV 60°/s | T1 × Grade I interaction | -1.19 | 1.84 | 62 | -0.65 | 0.521 | -4.86 to 2.49 |
| PEAK TQ/BW EV 60°/s | T2 × Grade I interaction | 1.81 | 1.99 | 62 | 0.91 | 0.367 | -2.17 to 5.79 |
| PEAK TQ/BW EV 60°/s | T1 × Grade II interaction | -0.78 | 1.75 | 62 | -0.44 | 0.659 | -4.28 to 2.73 |
| PEAK TQ/BW EV 60°/s | T2 × Grade II interaction | -0.57 | 1.9 | 62 | -0.3 | 0.766 | -4.37 to 3.23 |
| PEAK TQ/BW EV 60°/s | PRICE vs PEACE and LOVE | 1.98 | 1.31 | 60 | 1.51 | 0.135 | -0.64 to 4.60 |
| PEAK TQ/BW EV 60°/s | Age | 0.08 | 0.43 | 60 | 0.19 | 0.854 | -0.78 to 0.94 |
| PEAK TQ/BW IN 120°/s | Intercept | -4.09 | 7.14 | 62.14 | -0.57 | 0.569 | -18.37 to 10.19 |
| PEAK TQ/BW IN 120°/s | Grade I vs Grade III | 0.76 | 2.35 | 64.98 | 0.32 | 0.747 | -3.93 to 5.46 |
| PEAK TQ/BW IN 120°/s | Grade II vs Grade III | -2 | 2.16 | 62.01 | -0.92 | 0.359 | -6.31 to 2.32 |
| PEAK TQ/BW IN 120°/s | T1 vs T3 | 4.74 | 1.44 | 62.58 | 3.29 | 0.002 | 1.86 to 7.61 |
| PEAK TQ/BW IN 120°/s | T2 vs T3 | 2.03 | 0.91 | 62.01 | 2.24 | 0.028 | 0.22 to 3.85 |
| PEAK TQ/BW IN 120°/s | T1 × Grade I interaction | -2.58 | 2.68 | 61.96 | -0.96 | 0.339 | -7.93 to 2.77 |
| PEAK TQ/BW IN 120°/s | T2 × Grade I interaction | 1.22 | 1.7 | 62.01 | 0.72 | 0.476 | -2.17 to 4.60 |
| PEAK TQ/BW IN 120°/s | T1 × Grade II interaction | -0.82 | 2.56 | 61.98 | -0.32 | 0.750 | -5.93 to 4.29 |
| PEAK TQ/BW IN 120°/s | T2 × Grade II interaction | -0.57 | 1.62 | 62.01 | -0.35 | 0.728 | -3.80 to 2.67 |
| PEAK TQ/BW IN 120°/s | PRICE vs PEACE and LOVE | -0.32 | 1.4 | 60.2 | -0.23 | 0.818 | -3.13 to 2.48 |
| PEAK TQ/BW IN 120°/s | Age | 0.3 | 0.46 | 61.05 | 0.64 | 0.524 | -0.63 to 1.22 |
| PEAK TQ/BW EV 120°/s | Intercept | 3.36 | 5.36 | 61.97 | 0.63 | 0.533 | -7.35 to 14.07 |
| PEAK TQ/BW EV 120°/s | Grade I vs Grade III | -0.36 | 1.71 | 63.89 | -0.21 | 0.837 | -3.78 to 3.07 |
| PEAK TQ/BW EV 120°/s | Grade II vs Grade III | -1.69 | 1.57 | 61.1 | -1.08 | 0.285 | -4.83 to 1.45 |
| PEAK TQ/BW EV 120°/s | T1 vs T3 | 2.57 | 0.9 | 62.12 | 2.87 | 0.006 | 0.78 to 4.36 |
| PEAK TQ/BW EV 120°/s | T2 vs T3 | -0.35 | 0.69 | 62 | -0.51 | 0.610 | -1.74 to 1.03 |
| PEAK TQ/BW EV 120°/s | T1 × Grade I interaction | -0.97 | 1.66 | 61.65 | -0.59 | 0.560 | -4.30 to 2.35 |
| PEAK TQ/BW EV 120°/s | T2 × Grade I interaction | 1.68 | 1.29 | 62 | 1.29 | 0.200 | -0.91 to 4.26 |
| PEAK TQ/BW EV 120°/s | T1 × Grade II interaction | -0.89 | 1.59 | 61.67 | -0.56 | 0.576 | -4.07 to 2.28 |
| PEAK TQ/BW EV 120°/s | T2 × Grade II interaction | 0.67 | 1.24 | 62 | 0.54 | 0.591 | -1.80 to 3.14 |
| PEAK TQ/BW EV 120°/s | PRICE vs PEACE and LOVE | 0.22 | 1.05 | 60.18 | 0.21 | 0.836 | -1.89 to 2.32 |
| PEAK TQ/BW EV 120°/s | Age | -0.14 | 0.35 | 61.11 | -0.4 | 0.691 | -0.84 to 0.56 |
| ROM 60°/s | Intercept | -11.23 | 9.54 | 60.79 | -1.18 | 0.244 | -30.31 to 7.85 |
| ROM 60°/s | Grade I vs Grade III | -4.77 | 2.92 | 62.85 | -1.63 | 0.108 | -10.62 to 1.07 |
| ROM 60°/s | Grade II vs Grade III | -3.79 | 2.67 | 60.48 | -1.42 | 0.161 | -9.13 to 1.55 |
| ROM 60°/s | T1 vs T3 | 6.27 | 2.08 | 62 | 3.02 | 0.004 | 2.12 to 10.42 |
| ROM 60°/s | T2 vs T3 | 2.25 | 1.77 | 62.04 | 1.27 | 0.208 | -1.29 to 5.78 |
| ROM 60°/s | T1 × Grade I interaction | 2.22 | 3.88 | 62 | 0.57 | 0.569 | -5.54 to 9.98 |
| ROM 60°/s | T2 × Grade I interaction | 3.64 | 3.31 | 62.04 | 1.1 | 0.275 | -2.97 to 10.26 |
| ROM 60°/s | T1 × Grade II interaction | 0.37 | 3.7 | 62 | 0.1 | 0.920 | -7.03 to 7.78 |
| ROM 60°/s | T2 × Grade II interaction | -3.29 | 3.16 | 62.04 | -1.04 | 0.301 | -9.61 to 3.02 |
| ROM 60°/s | PRICE vs PEACE and LOVE | -1.93 | 1.88 | 60.07 | -1.03 | 0.309 | -5.70 to 1.83 |
| ROM 60°/s | Age | 0.76 | 0.62 | 60.07 | 1.23 | 0.224 | -0.48 to 2.00 |
| ROM 120°/s | Intercept | -12.63 | 9.49 | 61.31 | -1.33 | 0.188 | -31.61 to 6.34 |
| ROM 120°/s | Grade I vs Grade III | -4.74 | 3.01 | 63.53 | -1.58 | 0.120 | -10.75 to 1.26 |
| ROM 120°/s | Grade II vs Grade III | -4.43 | 2.75 | 60.83 | -1.61 | 0.113 | -9.93 to 1.07 |
| ROM 120°/s | T1 vs T3 | 5.92 | 2.23 | 59.36 | 2.66 | 0.010 | 1.46 to 10.38 |
| ROM 120°/s | T2 vs T3 | 2.12 | 1.95 | 62.03 | 1.09 | 0.281 | -1.78 to 6.02 |
| ROM 120°/s | T1 × Grade I interaction | 4.1 | 4.14 | 58.74 | 0.99 | 0.327 | -4.20 to 12.39 |
| ROM 120°/s | T2 × Grade I interaction | 3.12 | 3.65 | 62.03 | 0.86 | 0.396 | -4.17 to 10.41 |
| ROM 120°/s | T1 × Grade II interaction | 0.25 | 3.96 | 58.76 | 0.06 | 0.950 | -7.67 to 8.17 |
| ROM 120°/s | T2 × Grade II interaction | -3.14 | 3.48 | 62.03 | -0.9 | 0.370 | -10.10 to 3.82 |
| ROM 120°/s | PRICE vs PEACE and LOVE | -2.2 | 1.87 | 60.02 | -1.18 | 0.245 | -5.94 to 1.54 |
| ROM 120°/s | Age | 0.87 | 0.62 | 60.44 | 1.4 | 0.165 | -0.37 to 2.10 |
| YBT-CS | Intercept | -2.38 | 3.75 | 60.46 | -0.63 | 0.528 | -9.87 to 5.12 |
| YBT-CS | Grade I vs Grade III | -2.34 | 1.1 | 62.39 | -2.13 | 0.037 | -4.53 to -0.14 |
| YBT-CS | Grade II vs Grade III | -1.1 | 1 | 60.76 | -1.1 | 0.276 | -3.09 to 0.90 |
| YBT-CS | T1 vs T3 | 1.58 | 0.76 | 62.02 | 2.07 | 0.043 | 0.05 to 3.11 |
| YBT-CS | T2 vs T3 | 0.25 | 0.81 | 62 | 0.31 | 0.759 | -1.36 to 1.86 |
| YBT-CS | T1 × Grade I interaction | 0.04 | 1.43 | 62.02 | 0.03 | 0.980 | -2.82 to 2.89 |
| YBT-CS | T2 × Grade I interaction | -1.02 | 1.51 | 62 | -0.67 | 0.503 | -4.04 to 2.00 |
| YBT-CS | T1 × Grade II interaction | 2.08 | 1.36 | 62.02 | 1.53 | 0.132 | -0.64 to 4.81 |
| YBT-CS | T2 × Grade II interaction | 0.36 | 1.44 | 62 | 0.25 | 0.802 | -2.52 to 3.24 |
| YBT-CS | PRICE vs PEACE and LOVE | 0.94 | 0.74 | 60.03 | 1.27 | 0.210 | -0.54 to 2.42 |
| YBT-CS | Age | 0.22 | 0.24 | 60.03 | 0.92 | 0.362 | -0.26 to 0.71 |

**Supplementary Table S3.** Omnibus tests of fixed effects from covariate-adjusted linear mixed models of injured-limb functional outcomes. Fixed effects: Severity, Time, Severity × Time, age, and original treatment allocation. Repeated observations were modeled within participant using an unstructured covariance matrix; REML estimation and Satterthwaite degrees of freedom were used.

| **Outcome** | **Fixed effect** | **Numerator df** | **Denominator df** | **F** | **P** |
| --- | --- | --- | --- | --- | --- |
| PEAK TQ/BW IN 60°/s | Severity | 2.00 | 61.23 | 2.28 | 0.111 |
| PEAK TQ/BW IN 60°/s | Time | 2.00 | 62.04 | 31.47 | <0.001 |
| PEAK TQ/BW IN 60°/s | Severity × Time | 4.00 | 62.04 | 0.12 | 0.976 |
| PEAK TQ/BW IN 60°/s | Age | 1.00 | 60.08 | 0.47 | 0.495 |
| PEAK TQ/BW IN 60°/s | Treatment | 1.00 | 60.08 | 0.36 | 0.550 |
| PEAK TQ/BW EV 60°/s | Severity | 2.00 | 61.19 | 0.67 | 0.516 |
| PEAK TQ/BW EV 60°/s | Time | 2.00 | 62.00 | 28.53 | <0.001 |
| PEAK TQ/BW EV 60°/s | Severity × Time | 4.00 | 62.00 | 0.69 | 0.603 |
| PEAK TQ/BW EV 60°/s | Age | 1.00 | 60.00 | 1.89 | 0.175 |
| PEAK TQ/BW EV 60°/s | Treatment | 1.00 | 60.00 | 1.23 | 0.271 |
| PEAK TQ/BW IN 120°/s | Severity | 2.00 | 61.21 | 0.54 | 0.584 |
| PEAK TQ/BW IN 120°/s | Time | 2.00 | 61.87 | 24.65 | <0.001 |
| PEAK TQ/BW IN 120°/s | Severity × Time | 4.00 | 61.91 | 0.31 | 0.869 |
| PEAK TQ/BW IN 120°/s | Age | 1.00 | 61.11 | 0.00 | 0.945 |
| PEAK TQ/BW IN 120°/s | Treatment | 1.00 | 60.03 | 1.11 | 0.296 |
| PEAK TQ/BW EV 120°/s | Severity | 2.00 | 60.34 | 0.61 | 0.544 |
| PEAK TQ/BW EV 120°/s | Time | 2.00 | 61.87 | 38.00 | <0.001 |
| PEAK TQ/BW EV 120°/s | Severity × Time | 4.00 | 61.91 | 0.76 | 0.554 |
| PEAK TQ/BW EV 120°/s | Age | 1.00 | 60.53 | 0.61 | 0.438 |
| PEAK TQ/BW EV 120°/s | Treatment | 1.00 | 59.96 | 0.85 | 0.360 |
| ROM 60°/s | Severity | 2.00 | 60.81 | 0.21 | 0.813 |
| ROM 60°/s | Time | 2.00 | 62.02 | 24.13 | <0.001 |
| ROM 60°/s | Severity × Time | 4.00 | 62.02 | 1.24 | 0.303 |
| ROM 60°/s | Age | 1.00 | 60.05 | 10.50 | 0.002 |
| ROM 60°/s | Treatment | 1.00 | 60.05 | 0.02 | 0.878 |
| ROM 120°/s | Severity | 2.00 | 61.42 | 0.17 | 0.843 |
| ROM 120°/s | Time | 2.00 | 61.41 | 25.32 | <0.001 |
| ROM 120°/s | Severity × Time | 4.00 | 61.45 | 0.93 | 0.451 |
| ROM 120°/s | Age | 1.00 | 61.22 | 8.85 | 0.004 |
| ROM 120°/s | Treatment | 1.00 | 60.17 | 0.02 | 0.887 |
| YBT-CS | Severity | 2.00 | 61.19 | 2.28 | 0.110 |
| YBT-CS | Time | 2.00 | 62.03 | 19.45 | <0.001 |
| YBT-CS | Severity × Time | 4.00 | 62.03 | 0.37 | 0.831 |
| YBT-CS | Age | 1.00 | 60.06 | 2.14 | 0.149 |
| YBT-CS | Treatment | 1.00 | 60.06 | 1.49 | 0.227 |
|  |  |  |  |  |  |

**Supplementary Table S4.** Fixed-effect parameter estimates from covariate-adjusted linear mixed models of injured-limb functional outcomes. Reference-coded fixed-effect estimates. Clinical coding: Severity Grade I, Grade II, Grade III; Time T1, T2, T3; original treatment allocation PRICE=1 and PEACE and LOVE=2. SPSS used zero-based internal coding in the parameter table, so [Treatment=0] corresponds to PRICE and [Treatment=1] (reference) corresponds to PEACE and LOVE.

| **Outcome** | **Parameter** | **Estimate** | **SE** | **df** | **t** | **P** | **95% CI lower** | **95% CI upper** |
| --- | --- | --- | --- | --- | --- | --- | --- | --- |
| PEAK TQ/BW IN 60°/s | Grade I vs Grade III | 5.98 | 3.91 | 64.81 | 1.53 | 0.131 | -1.83 | 13.78 |
| PEAK TQ/BW IN 60°/s | Grade II vs Grade III | 0.77 | 3.63 | 61.31 | 0.21 | 0.832 | -6.49 | 8.04 |
| PEAK TQ/BW IN 60°/s | T1 vs T3 | -11.76 | 1.79 | 62.03 | -6.56 | <0.001 | -15.34 | -8.18 |
| PEAK TQ/BW IN 60°/s | T2 vs T3 | -5.41 | 1.08 | 62.00 | -5.01 | <0.001 | -7.56 | -3.25 |
| PEAK TQ/BW IN 60°/s | PRICE vs PEACE and LOVE | -1.17 | 1.95 | 60.08 | -0.60 | 0.550 | -5.07 | 2.73 |
| PEAK TQ/BW IN 60°/s | T1 × Grade I interaction | 0.04 | 3.35 | 62.03 | 0.01 | 0.990 | -6.66 | 6.74 |
| PEAK TQ/BW IN 60°/s | T2 × Grade I interaction | 0.73 | 2.02 | 62.00 | 0.36 | 0.719 | -3.30 | 4.76 |
| PEAK TQ/BW IN 60°/s | T1 × Grade II interaction | 1.48 | 3.20 | 62.03 | 0.46 | 0.645 | -4.91 | 7.88 |
| PEAK TQ/BW IN 60°/s | T2 × Grade II interaction | 0.61 | 1.93 | 62.00 | 0.32 | 0.751 | -3.23 | 4.46 |
| PEAK TQ/BW IN 60°/s | Age | 0.44 | 0.64 | 60.08 | 0.69 | 0.495 | -0.84 | 1.72 |
| PEAK TQ/BW EV 60°/s | Grade I vs Grade III | 2.74 | 2.88 | 65.49 | 0.95 | 0.345 | -3.01 | 8.48 |
| PEAK TQ/BW EV 60°/s | Grade II vs Grade III | 0.99 | 2.65 | 62.22 | 0.37 | 0.710 | -4.31 | 6.29 |
| PEAK TQ/BW EV 60°/s | T1 vs T3 | -7.03 | 1.05 | 62.00 | -6.72 | <0.001 | -9.12 | -4.94 |
| PEAK TQ/BW EV 60°/s | T2 vs T3 | -3.47 | 0.95 | 62.00 | -3.64 | 0.001 | -5.37 | -1.56 |
| PEAK TQ/BW EV 60°/s | PRICE vs PEACE and LOVE | -1.82 | 1.64 | 60.00 | -1.11 | 0.271 | -5.10 | 1.46 |
| PEAK TQ/BW EV 60°/s | T1 × Grade I interaction | 0.00 | 1.96 | 62.00 | 0.00 | 0.998 | -3.91 | 3.91 |
| PEAK TQ/BW EV 60°/s | T2 × Grade I interaction | -0.78 | 1.78 | 62.00 | -0.44 | 0.664 | -4.34 | 2.79 |
| PEAK TQ/BW EV 60°/s | T1 × Grade II interaction | 2.35 | 1.87 | 62.00 | 1.26 | 0.213 | -1.38 | 6.08 |
| PEAK TQ/BW EV 60°/s | T2 × Grade II interaction | -0.13 | 1.70 | 62.00 | -0.08 | 0.940 | -3.53 | 3.27 |
| PEAK TQ/BW EV 60°/s | Age | 0.74 | 0.54 | 60.00 | 1.37 | 0.175 | -0.34 | 1.82 |
| PEAK TQ/BW IN 120°/s | Grade I vs Grade III | 1.23 | 3.27 | 65.10 | 0.38 | 0.707 | -5.30 | 7.76 |
| PEAK TQ/BW IN 120°/s | Grade II vs Grade III | -2.16 | 3.03 | 61.60 | -0.71 | 0.480 | -8.22 | 3.91 |
| PEAK TQ/BW IN 120°/s | T1 vs T3 | -9.88 | 1.63 | 62.72 | -6.06 | <0.001 | -13.13 | -6.62 |
| PEAK TQ/BW IN 120°/s | T2 vs T3 | -5.12 | 0.97 | 62.01 | -5.28 | <0.001 | -7.05 | -3.18 |
| PEAK TQ/BW IN 120°/s | T1 × Grade I interaction | 0.68 | 3.04 | 62.10 | 0.22 | 0.823 | -5.39 | 6.75 |
| PEAK TQ/BW IN 120°/s | T2 × Grade I interaction | 1.25 | 1.81 | 62.01 | 0.69 | 0.494 | -2.38 | 4.87 |
| PEAK TQ/BW IN 120°/s | T1 × Grade II interaction | 2.13 | 2.90 | 62.12 | 0.74 | 0.465 | -3.66 | 7.93 |
| PEAK TQ/BW IN 120°/s | T2 × Grade II interaction | 1.66 | 1.73 | 62.01 | 0.96 | 0.341 | -1.80 | 5.12 |
| PEAK TQ/BW IN 120°/s | PRICE vs PEACE and LOVE | -1.75 | 1.66 | 60.03 | -1.06 | 0.296 | -5.08 | 1.57 |
| PEAK TQ/BW IN 120°/s | Age | -0.04 | 0.55 | 61.11 | -0.07 | 0.945 | -1.14 | 1.06 |
| PEAK TQ/BW EV 120°/s | Grade I vs Grade III | 2.17 | 2.20 | 63.65 | 0.99 | 0.326 | -2.22 | 6.57 |
| PEAK TQ/BW EV 120°/s | Grade II vs Grade III | 1.01 | 2.01 | 61.10 | 0.50 | 0.618 | -3.01 | 5.03 |
| PEAK TQ/BW EV 120°/s | T1 vs T3 | -6.11 | 0.83 | 61.81 | -7.37 | <0.001 | -7.77 | -4.45 |
| PEAK TQ/BW EV 120°/s | T2 vs T3 | -2.24 | 0.64 | 62.00 | -3.48 | 0.001 | -3.52 | -0.95 |
| PEAK TQ/BW EV 120°/s | T1 × Grade I interaction | 0.28 | 1.54 | 61.37 | 0.18 | 0.855 | -2.80 | 3.36 |
| PEAK TQ/BW EV 120°/s | T2 × Grade I interaction | -1.27 | 1.20 | 62.00 | -1.06 | 0.295 | -3.67 | 1.13 |
| PEAK TQ/BW EV 120°/s | T1 × Grade II interaction | 1.74 | 1.47 | 61.39 | 1.18 | 0.242 | -1.20 | 4.68 |
| PEAK TQ/BW EV 120°/s | T2 × Grade II interaction | -0.33 | 1.15 | 62.00 | -0.28 | 0.778 | -2.62 | 1.97 |
| PEAK TQ/BW EV 120°/s | PRICE vs PEACE and LOVE | -1.28 | 1.38 | 59.96 | -0.92 | 0.360 | -4.05 | 1.49 |
| PEAK TQ/BW EV 120°/s | Age | 0.36 | 0.46 | 60.53 | 0.78 | 0.438 | -0.56 | 1.27 |
| ROM 60°/s | Grade I vs Grade III | 0.39 | 4.77 | 62.63 | 0.08 | 0.935 | -9.14 | 9.92 |
| ROM 60°/s | Grade II vs Grade III | 0.83 | 4.36 | 60.00 | 0.19 | 0.850 | -7.89 | 9.55 |
| ROM 60°/s | T1 vs T3 | -9.30 | 2.43 | 62.00 | -3.83 | <0.001 | -14.16 | -4.44 |
| ROM 60°/s | T2 vs T3 | -3.38 | 1.97 | 62.02 | -1.72 | 0.091 | -7.32 | 0.55 |
| ROM 60°/s | T1 × Grade I interaction | -4.84 | 4.54 | 62.00 | -1.06 | 0.291 | -13.92 | 4.25 |
| ROM 60°/s | T2 × Grade I interaction | -3.90 | 3.68 | 62.02 | -1.06 | 0.294 | -11.25 | 3.46 |
| ROM 60°/s | T1 × Grade II interaction | -7.49 | 4.34 | 62.00 | -1.73 | 0.089 | -16.16 | 1.18 |
| ROM 60°/s | T2 × Grade II interaction | -0.06 | 3.51 | 62.02 | -0.02 | 0.986 | -7.08 | 6.96 |
| ROM 60°/s | PRICE vs PEACE and LOVE | -0.47 | 3.01 | 60.05 | -0.15 | 0.878 | -6.50 | 5.57 |
| ROM 60°/s | Age | -3.22 | 0.99 | 60.05 | -3.24 | 0.002 | -5.20 | -1.23 |
| ROM 120°/s | Grade I vs Grade III | 0.57 | 4.68 | 62.68 | 0.12 | 0.904 | -8.79 | 9.93 |
| ROM 120°/s | Grade II vs Grade III | 1.40 | 4.28 | 60.05 | 0.33 | 0.745 | -7.17 | 9.96 |
| ROM 120°/s | T1 vs T3 | -10.16 | 2.56 | 61.84 | -3.97 | <0.001 | -15.27 | -5.04 |
| ROM 120°/s | T2 vs T3 | -4.33 | 2.18 | 62.03 | -1.98 | 0.052 | -8.69 | 0.04 |
| ROM 120°/s | T1 × Grade I interaction | -6.09 | 4.76 | 61.26 | -1.28 | 0.205 | -15.60 | 3.41 |
| ROM 120°/s | T2 × Grade I interaction | -3.13 | 4.09 | 62.03 | -0.77 | 0.447 | -11.30 | 5.04 |
| ROM 120°/s | T1 × Grade II interaction | -6.51 | 4.54 | 61.28 | -1.43 | 0.157 | -15.58 | 2.57 |
| ROM 120°/s | T2 × Grade II interaction | 0.59 | 3.90 | 62.03 | 0.15 | 0.881 | -7.21 | 8.39 |
| ROM 120°/s | PRICE vs PEACE and LOVE | -0.42 | 2.94 | 60.17 | -0.14 | 0.887 | -6.30 | 5.47 |
| ROM 120°/s | Age | -2.90 | 0.97 | 61.22 | -2.98 | 0.004 | -4.85 | -0.95 |
| YBT-CS | Grade I vs Grade III | 7.47 | 2.90 | 62.84 | 2.58 | 0.012 | 1.68 | 13.27 |
| YBT-CS | Grade II vs Grade III | 4.26 | 2.64 | 60.90 | 1.61 | 0.112 | -1.02 | 9.53 |
| YBT-CS | T1 vs T3 | -7.16 | 2.29 | 62.00 | -3.12 | 0.003 | -11.75 | -2.57 |
| YBT-CS | T2 vs T3 | -2.37 | 0.88 | 62.06 | -2.69 | 0.009 | -4.14 | -0.61 |
| YBT-CS | T1 × Grade I interaction | -3.13 | 4.29 | 62.00 | -0.73 | 0.469 | -11.71 | 5.45 |
| YBT-CS | T2 × Grade I interaction | -1.19 | 1.65 | 62.06 | -0.72 | 0.475 | -4.48 | 2.11 |
| YBT-CS | T1 × Grade II interaction | -3.82 | 4.10 | 62.00 | -0.93 | 0.355 | -12.01 | 4.37 |
| YBT-CS | T2 × Grade II interaction | -0.73 | 1.57 | 62.06 | -0.47 | 0.642 | -3.88 | 2.41 |
| YBT-CS | PRICE vs PEACE and LOVE | -2.35 | 1.92 | 60.06 | -1.22 | 0.227 | -6.19 | 1.49 |
| YBT-CS | Age | 0.92 | 0.63 | 60.06 | 1.46 | 0.149 | -0.34 | 2.19 |

Note. Coefficients are conditional on the reference categories and other fixed effects in the model. They should be interpreted together with the omnibus Type III tests in Supplementary Table S1.

**Supplementary Table S5.** Omnibus tests of fixed effects from sensitivity models additionally adjusted for BMI. Fixed effects: Severity, Time, Severity × Time, age, original treatment allocation, and BMI; unstructured repeated covariance, REML estimation, Satterthwaite degrees of freedom.

| **Outcome** | **Fixed effect** | **Numerator df** | **Denominator df** | **F** | **P** |
| --- | --- | --- | --- | --- | --- |
| PEAK TQ/BW IN 60°/s | Severity | 2.00 | 59.10 | 2.50 | 0.091 |
| PEAK TQ/BW IN 60°/s | Time | 2.00 | 62.00 | 31.47 | <0.001 |
| PEAK TQ/BW IN 60°/s | Severity × Time | 4.00 | 62.00 | 0.12 | 0.976 |
| PEAK TQ/BW IN 60°/s | Age | 1.00 | 59.00 | 0.66 | 0.419 |
| PEAK TQ/BW IN 60°/s | Treatment | 1.00 | 59.00 | 0.51 | 0.477 |
| PEAK TQ/BW IN 60°/s | BMI | 1.00 | 59.00 | 2.02 | 0.161 |
| PEAK TQ/BW EV 60°/s | Severity | 2.00 | 60.24 | 0.65 | 0.523 |
| PEAK TQ/BW EV 60°/s | Time | 2.00 | 62.00 | 28.53 | <0.001 |
| PEAK TQ/BW EV 60°/s | Severity × Time | 4.00 | 62.00 | 0.69 | 0.603 |
| PEAK TQ/BW EV 60°/s | Age | 1.00 | 59.00 | 2.13 | 0.149 |
| PEAK TQ/BW EV 60°/s | Treatment | 1.00 | 59.00 | 1.30 | 0.260 |
| PEAK TQ/BW EV 60°/s | BMI | 1.00 | 59.00 | 0.83 | 0.365 |
| PEAK TQ/BW IN 120°/s | Severity | 2.00 | 59.69 | 0.71 | 0.497 |
| PEAK TQ/BW IN 120°/s | Time | 2.00 | 61.85 | 24.64 | <0.001 |
| PEAK TQ/BW IN 120°/s | Severity × Time | 4.00 | 61.89 | 0.31 | 0.869 |
| PEAK TQ/BW IN 120°/s | Age | 1.00 | 60.01 | 0.01 | 0.937 |
| PEAK TQ/BW IN 120°/s | Treatment | 1.00 | 59.00 | 1.36 | 0.248 |
| PEAK TQ/BW IN 120°/s | BMI | 1.00 | 58.75 | 1.74 | 0.192 |
| PEAK TQ/BW EV 120°/s | Severity | 2.00 | 59.26 | 0.59 | 0.555 |
| PEAK TQ/BW EV 120°/s | Time | 2.00 | 61.87 | 37.99 | <0.001 |
| PEAK TQ/BW EV 120°/s | Severity × Time | 4.00 | 61.92 | 0.76 | 0.555 |
| PEAK TQ/BW EV 120°/s | Age | 1.00 | 59.47 | 0.80 | 0.374 |
| PEAK TQ/BW EV 120°/s | Treatment | 1.00 | 58.97 | 0.94 | 0.335 |
| PEAK TQ/BW EV 120°/s | BMI | 1.00 | 58.84 | 1.00 | 0.321 |
| ROM 60°/s | Severity | 2.00 | 59.73 | 0.22 | 0.806 |
| ROM 60°/s | Time | 2.00 | 62.01 | 24.13 | <0.001 |
| ROM 60°/s | Severity × Time | 4.00 | 62.01 | 1.24 | 0.303 |
| ROM 60°/s | Age | 1.00 | 59.03 | 9.90 | 0.003 |
| ROM 60°/s | Treatment | 1.00 | 59.03 | 0.03 | 0.858 |
| ROM 60°/s | BMI | 1.00 | 59.03 | 0.36 | 0.550 |
| ROM 120°/s | Severity | 2.00 | 60.47 | 0.16 | 0.853 |
| ROM 120°/s | Time | 2.00 | 61.34 | 25.31 | <0.001 |
| ROM 120°/s | Severity × Time | 4.00 | 61.39 | 0.93 | 0.451 |
| ROM 120°/s | Age | 1.00 | 60.10 | 8.26 | 0.006 |
| ROM 120°/s | Treatment | 1.00 | 59.12 | 0.03 | 0.852 |
| ROM 120°/s | BMI | 1.00 | 58.87 | 0.94 | 0.337 |
| YBT-CS | Severity | 2.00 | 60.37 | 2.25 | 0.114 |
| YBT-CS | Time | 2.00 | 62.00 | 19.45 | <0.001 |
| YBT-CS | Severity × Time | 4.00 | 62.00 | 0.37 | 0.831 |
| YBT-CS | Age | 1.00 | 59.00 | 2.10 | 0.153 |
| YBT-CS | Treatment | 1.00 | 59.00 | 1.47 | 0.230 |
| YBT-CS | BMI | 1.00 | 59.00 | 0.01 | 0.937 |
